# Conductive Polypropylene Additive Manufacturing Feedstock: Application to Aqueous Electroanalysis and Unlocking Nonaqueous Electrochemistry and Electrosynthesis

**DOI:** 10.1021/acsami.4c12967

**Published:** 2024-10-02

**Authors:** David L. O. Ramos, Robert D. Crapnell, Ridho Asra, Elena Bernalte, Ana C. M. Oliveira, Rodrigo A. A. Muñoz, Eduardo M. Richter, Alan M. Jones, Craig E. Banks

**Affiliations:** † Faculty of Science and Engineering, Manchester Metropolitan University, Dalton Building, Chester Street, Manchester M1 5GD, Great Britain; ‡ Institute of Chemistry, Federal University of Uberlândia, Uberlândia, Minas Gerais 38400-902, Brazil; § School of Pharmacy, University of Birmingham, Edgbaston, Birmingham B15 2TT, United Kingdom

**Keywords:** additive manufacturing, electroanalysis, organic electrochemistry, electrosynthesis, chlorpromazine

## Abstract

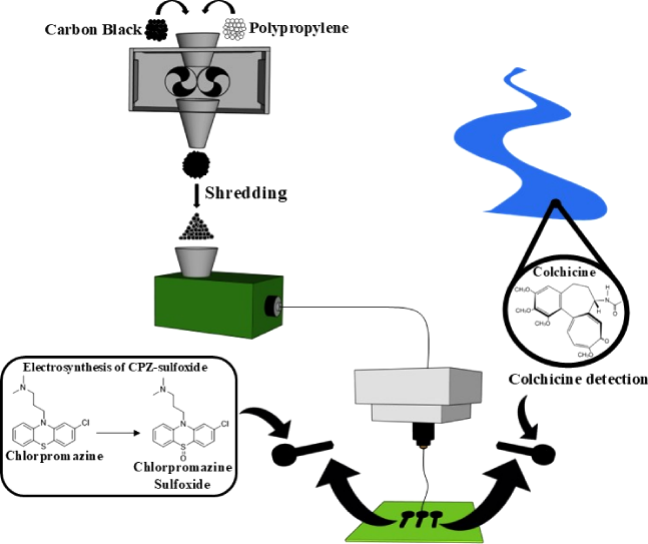

Additive manufacturing electrochemistry is an ever-expanding field; however, it is limited to aqueous environments due to the conductive filaments currently available. Herein, the production of a conductive poly(propylene) filament, which unlocks the door to organic electrochemistry and electrosynthesis, is reported. A filament with 40 wt % carbon black possessed enhanced thermal stability, excellent low-temperature flexibility, and high conductivity. The filament produced highly reproducible additive manufactured electrodes that were electrochemically characterized, showing a *k*^0^ of 2.00 ± 0.04 × 10^–3^ cm s^–1^. This material was then applied to three separate electrochemical applications. First, the electroanalytical sensing of colchicine within environmental waters, where a limit of detection of 10 nM was achieved before being applied to tap, bottled, and river water. Second, the electrodes were stable in organic solvents for 100 cyclic voltammograms and 15 days. Finally, these were applied toward an electrosynthetic reaction of chlorpromazine, where the electrodes were stable for 24-h experiments, outperforming a glassy carbon electrode, and were able to be reused while maintaining a good electrochemical performance. This material can revolutionize the field of additive manufacturing electrochemistry and expand research into a variety of new fields.

## Introduction

Additive manufacturing (also known as 3D printing) as a technology has been around for decades, with the first working machine being created by Charles W. Hull in 1984. With the ever-improving development of technology and lowering of prices, this manufacturing technique has exploded into various applications in recent years.^1^ One additive manufacturing technology that has seen significant uptake due to its low cost of entry is Fused Filament Fabrication (FFF, also known as Fused Deposition Modeling or FDM). This functions through the deposition of thermoplastic material in consecutive, thin-layered cross sections to produce a final 3-dimensional object. This form of manufacturing has allowed users to produce bespoke and complex part geometries with high degrees of customizability, low production run times and costs, and significantly lower waste production. Coupling these advantages to the global connectivity of additive manufacturing, whereby files can be designed and sent anywhere in the world for production, and the low cost of FFF printers and filament types, it is no wonder that it is becoming a staple technique within research environments.

One such research environment is within electrochemical laboratories, where, through the availability of inexpensive nonconductive and conductive filament, FFF has been used to create bespoke cells and accessories,^2^ equipment,^3^ and of course working electrodes.^4^ The only commercially available conductive filaments used in the literature for FFF printing all utilize poly(lactic acid) (PLA) as the base polymer, with carbon nanomaterials incorporated to induce conductivity. Although various efforts have been made to improve the performance of additive-manufactured electrodes printed from the commercially available conductive filament such as “activation,”^5,6^ which essentially removes surface polymer, changing the printing parameters,^7−10^ and surface modification,^11,12^ they remain substantially below the levels required to be competitive commercially. As such, research groups have recently taken to producing their own bespoke conductive filaments, allowing them to significantly increase the conductivities.^13^ While using PLA, or in many cases recycled PLA,^14^ researchers have managed to include various carbon materials, such as carbon black,^15^ graphite,^13^ and multiwalled carbon nanotubes,^16^ at significantly higher loadings than seen in commercially available filament. This has allowed for the production of electroanalytical sensors,^17,18^ energy storage devices,^19^ and biosensors^14^ that show performance that would be beneficial within commercial applications.

With these new bespoke filaments, conductivity and electrochemical performance were no longer the issues holding back their uptake into commercial products or new fields; however, the polymer is. Although PLA is ubiquitous with FFF due to its ease of printing, even with significant levels of filler, it has extremely poor chemical stability^20^ that essentially limits its application within the field to aqueous electrochemistry. Even within this field, it remains unsuitable for widespread use as it suffers from significant solution ingress,^21^ rendering any item single-use for fear of contaminating future samples. The use of single-use items, especially within electroanalytical sensors, is not uncommon; however, PLA is only biodegradable within industrial settings, for which there is not the infrastructure to ensure compliance with the United Nations Sustainable Development Goal 12—“Responsible Consumption and Production”. To overcome some of these issues, researchers have recently reported the production of conductive recycled poly(ethylene terephthalate glycol) (PETg), another polymer used regularly within FFF.^22^ PETg offers better chemical stability and improved mechanical properties over PLA. In a recent work, it was shown that electrodes printed from conductive PETg could be sterilized in both ethanol^22^ and under UV light, without exhibiting a reduction in electroanalytical performance, unlike similar PLA composites, offering a promising way into the healthcare markets. Additionally, due to the reduction in solution ingress for PETg, the working electrodes were able to be used up to 10 times before a deterioration in signal was observed, meaning significantly less waste would be produced.

Even so, the chemical stability properties of PETg still imply that only aqueous or alcoholic electrochemical systems can be explored.^20^ Unlocking the field of nonaqueous additive manufacturing electrochemistry has still not been accomplished. To this end, poly(propylene) (PP) has been identified as a material recently introduced within nonconductive FFF printing with excellent chemical stability properties in a wide range of solvents, including dichloromethane (DCM), chloroform, and dimethylformamide (DMF) among others. Expanding the arsenal of electrochemists to additive manufacturing electrochemistry within nonaqueous systems opens the door to bespoke and customizable electrode designs within fields such as nonaqueous electrosynthesis and energy storage, as well as widening the applications for electroanalytical chemists.

PP is a thermoplastic polymer member of the polyolefins and is partially crystalline and nonpolar. It is a common household plastic widely used to produce plastic furniture, low-friction plastic items, and packaging. It has found particular use within packaging for harsh chemicals such as cleaning products and bleaches and is often recommended for storing harsh chemical solutions in laboratory environments. Due to this chemical stability and the ability to print nonconductive PP using FFF, it has been identified as a potentially revolutionary base polymer for additive manufacturing electrochemistry.

Therefore, in this work, we look for the first time into the development of a high-performance conductive filament for FFF by combining PP with high loadings of carbon black (CB) as the conductive nanomaterial. Through optimizing the production and printing, as well as fully electrochemically characterizing the electrodes and applying them toward three new applications: (i) detection of the emerging contaminant colchicine (CCH) in water samples; (ii) detection of ferrocene as a model-molecule in nonaqueous solvents (acetonitrile, MeCN, dichloromethane, and dimethylformamide); and (iii) electrosynthesis of chlorpromazine metabolites in aprotic solvents such as dimethylformamide (DMF) and MeCN, we hope to unlock additive manufacturing electrochemistry within nonaqueous electrolytes and revolutionize the field.

## Results and Discussion

### Production and Characterization of Conductive Polypropylene Filament

**Figure 1:**
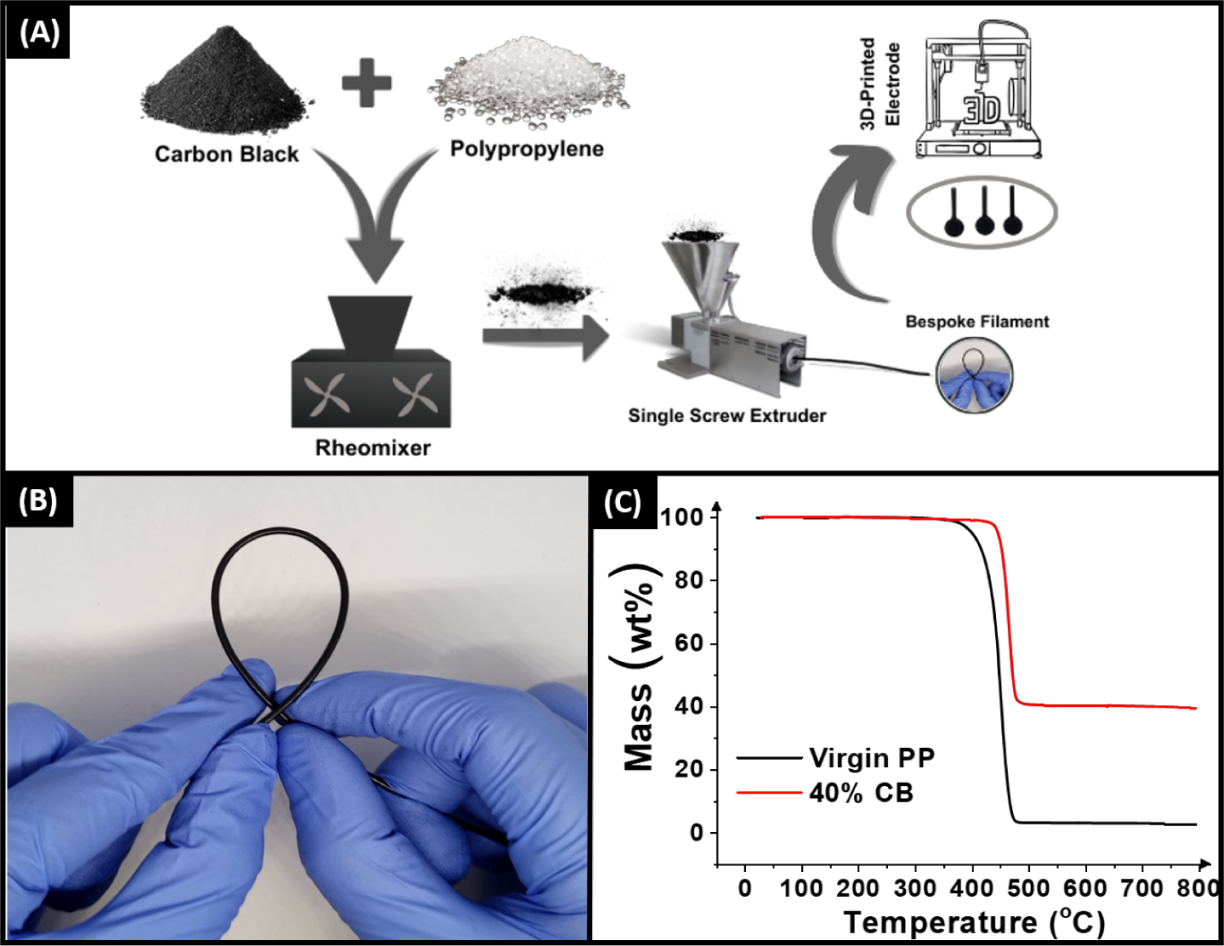
(A) Filament production scheme. (B) Photograph highlighting the low temperature flexibility of the 40 wt % CB filament. (C) Thermogravimetric analysis of the virgin PP pellets and the 40 wt % CB filament.

Initially, polymer compositions combining different amounts of carbon black (CB) with poly(propylene) (PP) were produced, where the CB was increased from 15 wt % to 40 wt %, with the amount of PP reduced by the same amount in turn. A scheme representing the production of the bespoke conductive PP filaments can be seen in Figure 1A. Briefly, the CB powder and PP pellets were mixed for 5 min at a temperature of 210 °C, after which the combined CB-filled polymer composite was collected. Compositions above 40 wt % CB failed to fully incorporate during the mixing process and were therefore not explored further. The collected polymer composites were then shredded and processed through a single screw extruder at 210 °C to produce the final filaments. In all cases, a filament with excellent low-temperature flexibility was obtained, as shown in Figures 1B and S1. When comparing the filaments, once the CB loading has exceeded 30 wt %, there is a noticeable reduction in the low-temperature flexibility. Even with this reduction, the 40 wt % CB filament still possesses excellent flexibility and allows for easy spooling and printing.

There are no reports of conductive PP filaments for use within additive manufacturing electrochemistry, and therefore, it is important to look elsewhere for benchmarks. To achieve this, we look toward both commercially available conductive PLA and bespoke reports in the literature of conductive PLA and PETg. These filaments all quote the bulk resistance obtained across a 10 cm portion of filament as an indication of how conductive they are. Each filament made using CB and PP was measured in this way, with the results presented in Table S1. As the amount of CB increased, the resistance of the filament was significantly decreased. The lowest resistance of 24.7 ± 3.0 Ω cm^–1^ was obtained for the 40 wt % CB filament, which is significantly lower than the commercially available PLA for which we obtained resistance values of 380 ± 70 Ω cm^–1^. The resistance of the 40 wt % CB/PP filament also offered excellent performance when compared to reports of bespoke filament within the literature, with values of 86.4 ± 5.4 Ω cm^–1^ obtained for a CB in PLA filament,^15^ 27.7 ± 0.9 Ω cm^–1^ for an optimized CB and graphite PLA,^18^ 24.3 ± 2.4 Ω cm^–1^ for a multiwalled carbon nanotube and CB PLA,^16^ and 71.0 ± 3.0 Ω cm^–1^ for the first reported conductive PETg.^22^ This indicates that the filament containing 40 wt % CB within PP is extremely conductive, while also not requiring any additional plasticizer found within the PLA filaments or the expensive mix of conductive carbons found within the PETg.

Thermogravimetric analysis (TGA) was performed on the 40 wt % bespoke CB/PP filament, along with the virgin PP pellets, to understand how the inclusion of these fillers affects the thermal stability of the polymer. This is an important characteristic with additive manufacturing for the intended end use. It can also provide accurate information about the actual levels of conductive filler loading within the filament. The resultant plot of wt % versus temperature can be seen in Figure 1C, where in both cases, the expected sigmoidal curve is obtained corresponding to the thermal degradation of PP. For the virgin PP pellets, the onset of degradation temperature was calculated to be 331 ± 3 °C, showing good agreement with the literature.^23^ Interestingly, when 40 wt % CB is integrated into the polymer, the onset temperature of degradation significantly increases to a value of 378 ± 2 °C. This indicates that the CB is providing some thermal stabilization for the filament, most likely due to creating a physical barrier to gas diffusion out of the polymer and effectively slowing the rate of decomposition.^24^ Through analyzing the stabilization of the curve after the degradation of PP, we can elucidate the exact amount of CB filler in the filament. Through subtracting the stabilization of virgin PP from the value for the 40 wt % sample, we calculate a result of 39 ± 2 wt %, which indicates that no material was lost during filament production.

**Figure 2:**
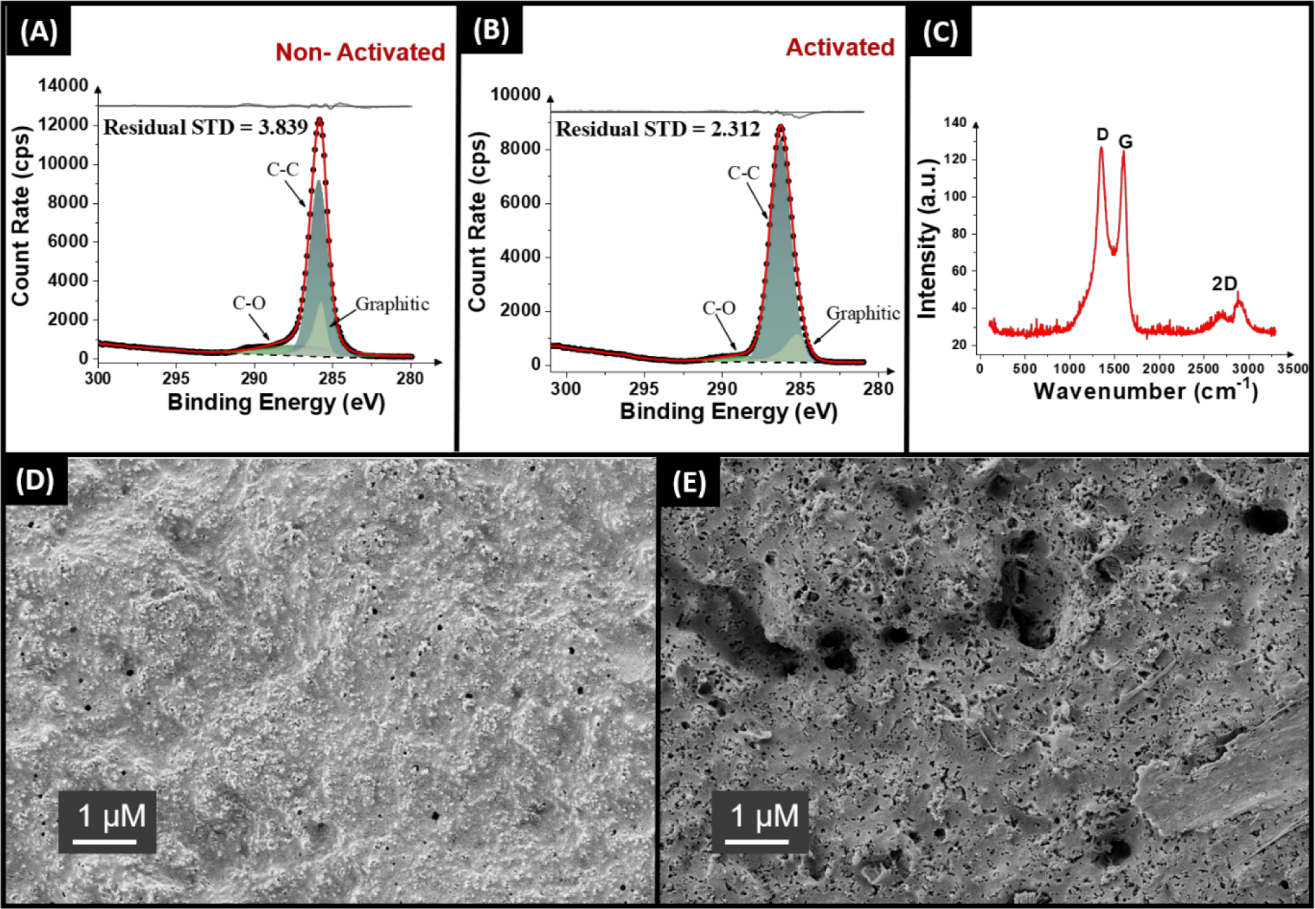
XPS C 1s spectra of the 40 wt % CB/PP electrodes (A) before and (B) after activation. (C) Raman spectra for the activated 40 wt % CB/PP electrode. SEM images of the 40 wt % CB/PP electrodes (D) before and (E) after activation.

After evaluation of the filament, it was used to print lollipop electrodes of identical dimensions as reported elsewhere,^15,22^ to allow for appropriate comparisons with the literature. For use within electrochemistry, most additively manufactured electrodes are activated, which removes the surface polymer from the electrode and exposes increased amounts of conductive carbon beneath. This is important, as gloving effects from the extrusion and printing processes often lead to the migration of a film of polymer to the outside of the object. The mechanical polishing and electrochemical activation with 0.5 M NaOH methods were tried, and it was found that electrochemical activation produced improved performance for these CB/PP electrodes. It is important to establish what effect this had on the surface of the additively manufactured electrodes; hence, they were subjected to XPS analysis. Figure 2A,B shows the C 1s spectra obtained for the as-printed and activated electrodes, respectively.

In both the nonactivated and activated samples, there are three peaks assigned to produce an appropriate fit within XPS spectra. In both cases, there is a large symmetric peak at 285.0 eV, which corresponds to the sp^3^ carbon bonding within PP and CB, respectively. When considering the overall proportions of PP and CB within this filament, a peak of significant intensity compared to the other required peaks is expected due to the chemical structure of PP. The other peaks required for the fitting arise from the presence of CB within the sample, namely C–O bonding on the surface and importantly an asymmetric peak at 284.5 eV assigned to the X-ray photoelectron emission of graphitic carbon.^25,26^ Interestingly, unlike in previous work with PLA^13,15−18^ and PETg,^22,27^ there is no significant increase in the graphitic carbon peak after the activation procedure is carried out. This indicates that there is not a significant enhancement in the amount of surface CB available, but the presence of the graphitic carbon peak indicates there is surface carbon available for electrochemistry.

To further examine the material, Raman spectroscopy was next performed (Figure 2C), where there are clearly defined peaks present at 1338, 1572, and 2680 cm^–1^. These peaks are attributed to the characteristic D-, G-, and 2D bands found within the Raman spectra for graphitic-like structures. Through calculating the *I*_D_/*I*_G_ ratio within the spectra of 1.01, the presence of CB on the surface can be confirmed. The findings through XPS and Raman are corroborated through SEM analysis of the samples, where the nonactivated electrode can be seen in Figure 2D and the activated electrode in Figure 2E. On the surface of the nonactivated electrode in Figure 2D, there is a smooth covering of polymer over the surface, as well as small circular objects extruding from the surface, which correspond to the CB particles. Additionally, small perforations in the polymer film can be seen, most likely due to the high levels of filler loading, placing a strain on the structure. Interestingly, when activated within aqueous 0.5 M NaOH (Figure 2E), the perforations are significantly increased in number and size. There is still a significant amount of surface polymer present, which is expected, as PP should be resistant to the solution used. However, clearly, the combination of NaOH and chronoamperometry (+1.4 V for 200 s, followed by −1.0 V for 200 s) causes expansion of potential weak points in the surface. This supports the findings from XPS, where although there is no increase in surface graphitic carbon, there is an increase in porosity of the electrode, meaning that increased amounts of molecules could reach the conductive carbon within the electrode. As such, we would expect to see an improvement in the electrochemical performance with the activated electrode.

### Electrochemical Characterization

**Figure 3:**
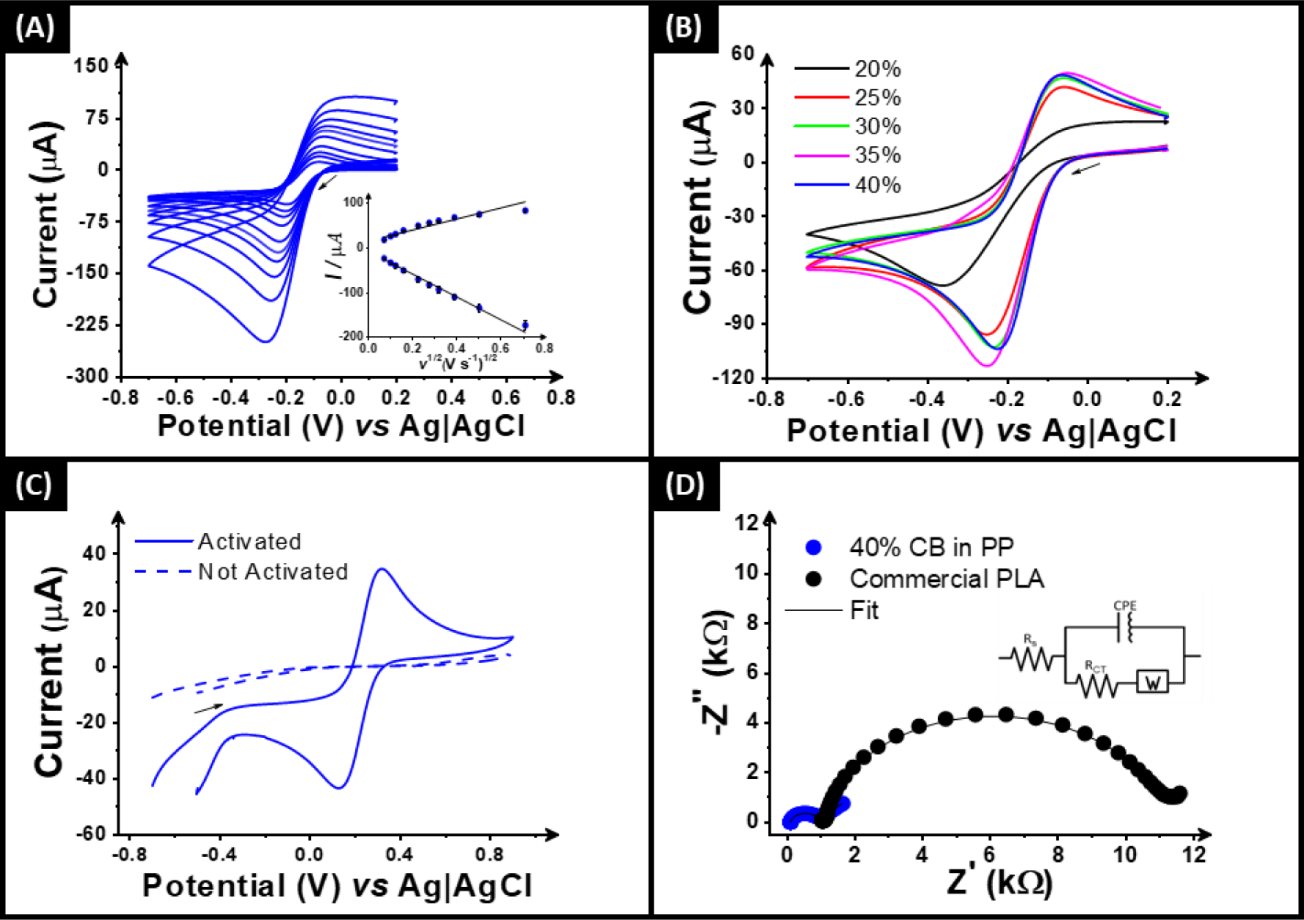
(A) Scan rate study (5–500 mV s^–1^) with [Ru(NH_3_)_6_]^3+^ (1 mM in 0.1 M KCl) performed in 40% CB in the PP electrode. Inset: the Randles–Ševčík plot. (B) CVs (50 mV s^–1^) of [Ru(NH_3_)_6_]^3+^ comparing the electrodes with different amounts of CB in polypropylene (20%, 25%, 30%, 35%, and 40% CB) and (C) CVs (25 mV s^–1^) of [Fe(CN)_6_]^4–^ (1 mM in 0.1 M KCl) with and without activation in 0.5 M NaOH solution using 40% CB in PP as the WE. (D) EIS Nyquist plots of [Fe(CN)_6_]^4–/3–^ comparing commercial conductive PLA filament with 40% CB in PP. Inset: the proposed equivalent circuit.

Once physicochemically characterized, it is important to benchmark the electrochemical performance of the 3D-printed electrode. This was achieved in the same ways as outlined previously,^15,16,22^ initially through scan rate studies against the near-ideal outer-sphere redox probe hexaamineruthenium(III) chloride ([Ru(NH_3_)_6_]^3+^). This allows for the best determination of the heterogeneous electron (charge) transfer rate constant (*k*^0^), as well as the real electrochemical surface area (*A*_e_); see Experimental Section.^28^ An example of the cyclic voltammograms (ν = 5–500 mV s^–1^) obtained through the scan rate study for the electrodes printed from the 40 wt % CB filament is presented in Figure 3A (inset with the associated Randles-Ševčík plots), and the others are described in Figure S2.

Figure 3B shows a comparison between the cyclic voltammograms obtained at 50 mV s^–1^ for the electrodes printed out of all the CB/PP filaments prepared in this work except 15 wt %, which gave no signal. It can be seen clearly that 20 wt % is completely inadequate for electrochemical applications; however, all other compositions present the expected redox response, with a reduction peak for [Ru(NH_3_)_6_]^3+^ at ∼−0.2 V. From the data collected, it was possible to calculate the average peak-to-peak separation (Δ*E*_p_), as well as the *k*^0^ and *A*_e_, which is summarized in Table 1.

**Table 1:** Electrochemical Characterization Data Calculated from the Scan Rate Studies in [Ru(NH_3_)_6_]^3+^ (1 mM in 0.1 M KCl), Including the Peak-to-Peak Separation (Δ*E*_p_), Heterogeneous Electron (Charge) Transfer Rate Constant (*k*^0^), as well as the Real Electrochemical Surface Area (*A*_e_) (n = 3)

Filament (% CB)	Δ*E*p (mV)	*k*^0^ (x10^–3^ cm s^–1^)	*A*_e_ (cm^2^)
15	-	-	-
20	270 ± 20	0.47 ± 0.05	15 ± 1
25	150 ± 17	1.40 ± 0.06	37 ± 3
30	140 ± 13	1.73 ± 0.04	44 ± 3
35	170 ± 45	1.21 ± 0.15	42 ± 4
40	120 ± 11	2.00 ± 0.04	45 ± 3

When inspecting Table ,1 increasing the carbon content of the filament improves the electrochemical performance in all three parameters. It should be noted that there is a decrease in performance for the 35 wt % sample, but the errors seen within this data set are also significantly larger than any of the others. Overall, for the 40 wt % CB filament, a Δ*E*_p_ of 120 ± 11 mV was measured, along with a calculated *k*^0^ of 2.00 ± 0.04 × 10^–3^ cm s^–1^ and an *A*_e_ of 45 ± 3 cm^2^. When comparing these findings to previous reports of bespoke conductive filaments, we see that it performs well. Previous reports of bespoke conductive PLA produced *k*^0^ values of 1.71 ± 0.22 × 10^–3^ cm s^–1^,^15^ 1.26 × 10^–3^ cm s^–1^,^13^ and 2.6 ± 0.1 × 10^–3^ cm s^–1^,^16^ which demonstrates that the 40 wt % CB/PP filament developed in this work is very competitive. Although comparison is made considering that the PP filament reported here contains 40 wt % CB compared to 25–35 wt % reported in PLA, it is important to remark that this is achieved without using any additional plasticizer during the filament making, whereas all the bespoke PLA reported using this production method required plasticizers to achieve the carbon loadings mentioned above. On the other hand, comparing to the first reports of conductive PETg filament that did not include plasticizer in the filament formulation, they produced *k*^0^ values of 0.88 × 10^–3^ cm s^–1^^22^ and 1.03 × 10^–3^ cm s^–1^^27^ respectively, which highlights the significant improvement in electrochemical performance achieved by the new CB/PP filament.

To further evaluate the electrochemical performance of the 40 wt % CB/PP filament, it was tested against the commonly used inner-sphere redox probe [Fe(CN)_6_]^4–^ (1 mM in 0.1 M KCl). Figure 3C presents the cyclic voltammograms (25 mV s^–1^) obtained using additively manufactured electrodes printed from the 40 wt % CB/PP filament before (dashed line) and after (solid line) electrochemical activation within 0.5 M NaOH. It can be clearly seen that there is no electrochemical response found before activation, which is due to the surface covering of PP on the electrode. Clearly, when compared back to the SEM images in Figure 2, the expansion of the pores through electrochemical activation within NaOH must allow significant access to the CB embedded within the electrode. It is the creation of these pores which helps the electrochemical performance for inner-sphere redox probes, since the XPS results confirmed that there was minimal change in the graphitic content on the surface. However, after activation, there are well-defined redox peaks corresponding to the [Fe(CN)6]^4–^ redox couple, displaying a Δ*E*_p_ of 185 mV, which highlights the excellent performance of the CB/PP electrode toward inner-sphere molecules. This was further tested through electrochemical impedance spectroscopy (EIS) using [Fe(CN)_6_]^4–/3–^ solution, with the Nyquist plots comparing the response of the activated 40 wt % CB/PP filament and the commonly used commercial conductive PLA filament shown in Figure 3D. It is demonstrated that the bespoke CB/PP filament significantly outperforms the commercial conductive PLA filament in terms of both the solution resistance (*R*_S_) and the charge-transfer resistance (*R*_CT_). Through appropriate circuit fitting, the *R*_S_ was calculated to be 181 ± 5 Ω and the *R*_CT_ was calculated to be 1.36 ± 0.10 kΩ, which again compare excellently to other bespoke conductive PLA filaments reported previously.^18^ Once electrochemically characterized, it was important to explore the potential applications for this new material as the base polymer offers significant improvements in chemical stability. We first look to test the electroanalytical performance of the material and then progress into applications that fully utilize this feature.

### Electrochemical Performance in Aqueous Solutions

#### Application toward the Electroanalysis of Colchicine (CCH) in Water Samples

**Figure 4:**
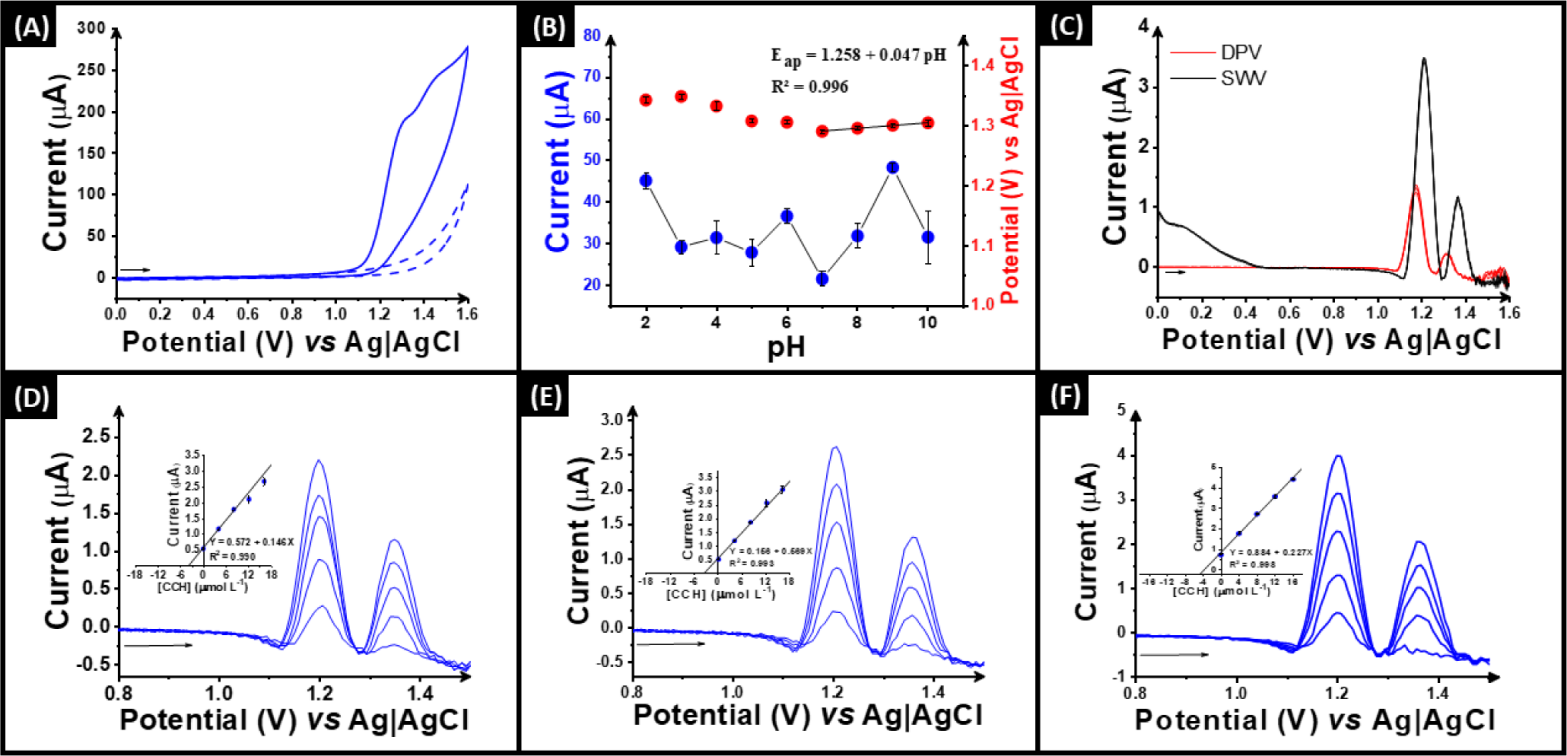
(A) Cyclic voltammogram of 1.0 mM CCH in 0.1 mol L^–1^ borate buffer, pH 9.0. (B) Plot of current and potential vs pH with 1.0 mM CCH in 0.1 M BR buffer performed at 40% CB in PP using LSV (scan rate 50 mV s^–1^, step potential 2 mV). (C) SWV and DPV performed at 40% CB in PP electrode (*n* = 3) in 0.1 mol L^–1^ borate buffer pH 9.0 using 20 μmol L^–1^ of CCH. SWV parameters: *a* = 20 mV, *f* = 25 Hz, and Δ*E* = 5 mV. DPV parameters: *a* = 25 mV and Δ*E* = 5 mV. SW voltammograms and standard addition calibration plots (inset) for the determination of CCH (4.0 to 20 μM) within (D) tap water, (E) bottled water, and (F) environmental river water samples.

To test the aqueous electroanalytical performance of the additively manufactured electrodes printed from the new 40 wt % CB/PP filament, we explored the detection of the colchicine (CCH) molecule in different water samples. This is a pharmaceutical compound that has been used for the treatment of Mediterranean fever, scleroderma, amyloidosis, and more recently COVID 19.^29−32^ It is a well-known and potent inhibitor of the inflammasome and impedes the release of interleukin-1.^33^ Previously, it had shown antiviral properties in response to Flaviviridae^34^ and mouse hepatitis.^35^ It was therefore proposed as a treatment for SARS-CoV-2; however, it was shown to be ineffective in this regard.^36^ Even so, CCH is still regularly used in some countries, leading to its detection within the environment. As such, analytical detection of this drug is an important challenge. First, an exploratory cyclic voltammetric study was performed in 1.0 mM CCH in 0.1 M borate buffer pH 9.0. According to the literature,^31,37^ two oxidation peaks are recognized in the voltammogram at +1.30 V and +1.45 V versus the Ag|AgCl reference electrode, which indicates that CCH can be detected using the CB/PP electrode. Second, a pH study was undertaken whereby linear sweep voltammetry (LSV) was utilized to observe the oxidation of CCH from pH 2.0 to 10.0 in BR-buffer (0.1 M). The values obtained for the peak current and peak potentials throughout the pH study can be seen in Figure 4B, where the maximum peak current was observed at pH 9. Based on this data, the slope obtained from plotting the potentials determined at the different pH was calculated as 0.047, which, according to the Nernst equation, suggests an oxidation process for CCH that involves an equal number of protons and electrons. This supports the mechanism by Bodoki et al.,^37^ shown in Figure S3. Note that LSV experiments displayed only one oxidation peak at +1.3 V vs Ag|AgCl, and that was therefore selected to perform the corresponding calculations. Following this, different pulse voltametric techniques were applied to determine the most sensitive technique for CCH quantification. As depicted in Figure 4C, square wave voltammetry (SWV) was compared to differential pulse voltammetry (DPV) for the detection of CCH in borate buffer at pH 9 (0.1 M). A clear improvement in peak current is observed for both oxidation peaks that can nowbe differentiated in the voltammogram, increasing from 1.40 ± 0.06 μA and 0.35 ± 0.01 μA for DPV to 3.67 ± 0.04 μA and 1.40 ± 0.04 μA for SWV. Therefore, SWV was selected for further analytical applications in real water samples.

Following the full optimization of the SWV technique (step = 8 mV, amplitude = 30 mV, and frequency = 30 s^–1^), a calibration plot for the detection of CCH was obtained in pH 9 borate buffer (0.1 M) (Figure S4). A linear relationship was observed for both oxidation peaks, allowing for the determination of CCH using indistinctively the first or the second peak. Specifically, the first peak gave a linear range between 2 and 50 μM, with a limit of detection (LoD) equal to 0.01 μM and a limit of quantification (LoQ) equal to 0.04 μM. In comparison, the second oxidation gave a linear range between 4 and 50 μM, with a LoD equal to 0.03 μM and an LoQ equal to 0.09 μM. Then, intra- and inter-day reproducibility experiments were performed for each peak, where in all cases, intra-day reproducibility was found to be below 5% and inter-day reproducibility was 7.4% for the first peak and 5.0% for the second peak. Furthermore, this system was tested for possible interferences against caffeine, glucose, arsenic, copper, lead, and chromium. Out of these interferents, it was found that only caffeine and lead showed potential interference toward CCH in water, where caffeine only hampered the second oxidation peak due to exhibiting close oxidation peak potentials in carbon-based electrodes and lead covered both peaks, which could be minimized by adding a complexing agent to remove this species from the real sample.

Finally, this system was then applied to the detection of CCH within tap (Figure 4D), bottled (Figure 4E), and environmental river water (Figure 4F) accordingly spiked with known concentration of CCH standard. For tap water, an LoD of 0.12 μM was obtained and a recovery of 98 ± 5% was found when using the first oxidation peak, which reported better detectability. Likewise, for bottled water, an LoD of 0.05 μM was obtained with a 91 ± 7% recovery, and for natural river water, an LoD of 0.03 μM and a recovery of 93 ± 3% was calculated. These results show the excellent analytical performance of this electrode and new material for CCH quantification in water samples, without additional pretreatment. When compared to other reports of sensors in the literature (Table 2), we can see that this additively manufactured electrode possesses an excellent and competitive LoD and is applied for the first time in environmental matrices.

**Table 2:** A Comparison of This Work with Other Reports on the Detection of CCH^a^

Electrode	Technique	Sample	LoD (μM)	**ref**
MIM/GaAuNPs/GCE	DPV	pharmaceutical/human serum	0.005	(38)
MWCNTs/CPE	DPV	pharmaceutical	0.008	(39)
GSPEs	DPV	pharmaceutical	0.01	(31)
MWCNTs/GCE	DPV	pharmaceutical/urine	0.015	(40)
BDD	FIA	pharmaceutical/urine	0.02	(41)
PoPD/SWNTs/GCE	DPV	pharmaceutical	0.03	(42)
SPE	BIA	pharmaceutical	0.1	(43)
CB/PLA	DPV	pharmaceutical/urine	0.1	(44)
GCE	DPV	pharmaceutical	0.2	(45)
BDD	DPV	pharmaceutical	0.3	(32)
40% CB in PP	SWV	tap, bottled, and river water	0.01	this work

a GSPEs: graphite-based screen-printed electrodes; CB: carbon black; PP: polypropyline; SPE: screen-printed electrode; GCE: glassy carbon electrode; PoPD/SWNTs/GCE: glassy carbon electrode modified with poly(*o*-phenylenediamine)/single-wall carbon nanotubes modified; CPE: carbon paste electrode; MWCNTs: multiwall carbon nanotubes; MIM/GaAuNPs/GCE: materials composed of graphene and gold nanoparticles and glassy carbon electrodes modified with molecularly printed membrane; PLA: polylactic acid; BDD: boron-doped diamond; CuO: copper oxide nanoparticules; CNF: carbon nanofiber composite; [Bim]FeCl_4_: 1-butyl-3-methylimidazolium tetrachloroferrate; DPV: differential pulse voltammetry; FIA: flow injection analysis; SWV: square wave voltammetry; BIA: batch injection analysis.

This section summarizes the excellent performance of this bespoke conductive PP filament for standard aqueous electroanalysis, eliminating the potential issues with water ingress reported previously for conductive PLA.^21^ In addition to this, PP has the added benefits of excellent chemical stability, which we will now explore to unlock completely new fields for additive manufacturing electrochemistry.

### Electrochemical Performance in Nonaqueous Solutions

#### Electrochemistry in Organic Solvents

**Figure 5:**
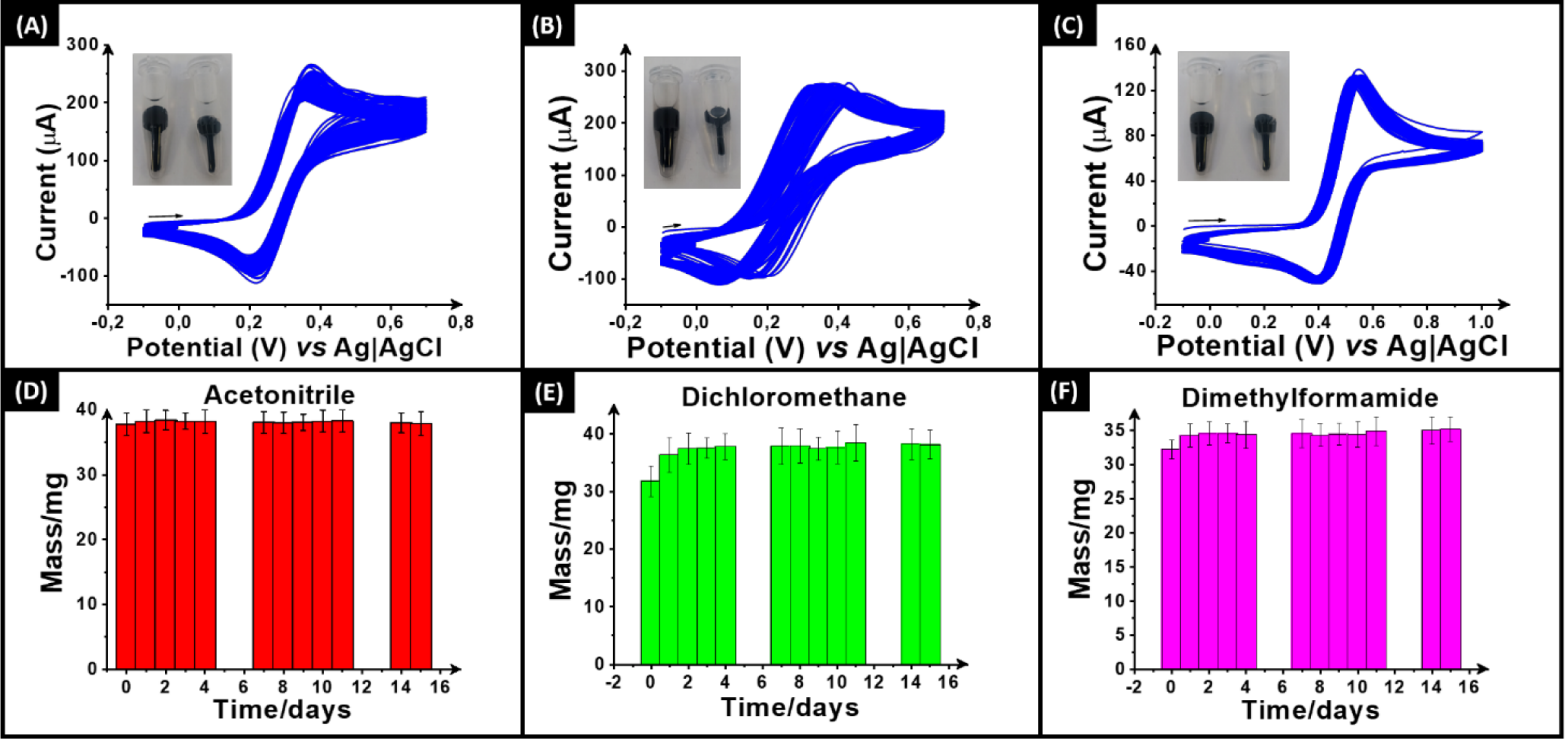
CVs studies (100 cycles) in (A) acetonitrile, (B) dichloromethane, and (C) dimethylformamide with 1.0 mM ferrocene in 100 mM *n*-tetrabutylammonium hexafluorophosphate with 40% CB in PP as the WE. Scan rate: 50 mV s^–1^. Stability of the electrodes in (D) acetonitrile, (E) dichloromethane, and (F) dimethylformamide was measured for 15 days.

After exploring the excellent aqueous electroanalytical performance of CB/PP, we now turn to explore the stability and electrochemical performance of the 40 wt % CB/PP filament in nonaqueous solvents. Figure 5A–C shows the electrochemical response of the activated additively manufactured electrodes toward ferrocene (1 mM in 0.1 M TBAF) in acetonitrile (MeCN), dichloromethane (DCM), and dimethylformamide (DMF). The inset in each figure is a photograph of an electrode printed from commercial conductive CB/PLA (left) and the bespoke 40 wt % CB/PP (right) in the associated solvent. The CB/PLA electrode was immersed for 30 s, and the CB/PP electrode was immersed for 60 min. In all cases, there is no visible change in the CB/PP electrode, whereas there is significant swelling in the CB/PLA electrode after only 30 s. As expected, this supports previous observations confirming that PLA is completely unsuitable for performing any electrochemistry in nonaqueous environments. On the other hand, the PP electrode offers excellent chemical stability and clearly allows for the collection of electrochemical data within these solvents. A continuous 100 scans of 1 mM ferrocene in cyclic voltammetry (50 mV s^–1^) within the same solvent using the same electrode were registered versus a silver wire *pseudo* reference electrode, as depicted in Figure 5A–C. Note that the use of a *pseudo* reference helps to explain some of the shifts in the measured peak potentials, but in all cases, the measured peak current remains relatively stable over the course of 100 scans, which highlights the exceptional stability of the CB/PP electrodes. In the case of DMF (Figure 5C), remarkable stability is observed in terms of both peak potential and current over the course of 100 scans.

To further test the chemical stability of the electrodes printed from the 40 wt % CB/PP filament in these solvents, they were submerged for 15 days and their masses measured at regular intervals. Figure 5D, E and F show the results for MeCN, DCM, and DMF, where an excellent stability in the measurements can be seen across the whole time frame. In the case of MeCN, there is almost no change throughout the 15 days, whereas for the DCM and DMF, there are initial increases in the measured masses, which indicate a small amount of solution ingress that stabilizes quickly. This is in agreement with work seen on the stability of printed PP within these two solvents,^20^ where they possess in general excellent stability but do show subtle changes in the surface microstructure after prolonged exposure. These results highlight the chemical stability of the material, which is important for enabling long-term use within these media. For other printable materials, submersion within these solvents can lead to the destruction of the polymer and failure of the printed part through various processes such as dissolution, disintegration, delamination, and swelling.^20^ After seeing the excellent stability of this material in these solvents, we now progress to look at an application that, before now, had been impossible for electrochemists using additive manufacturing.

### Application toward Electrosynthesis of Chlorpromazine Metabolites

**Figure 6:**
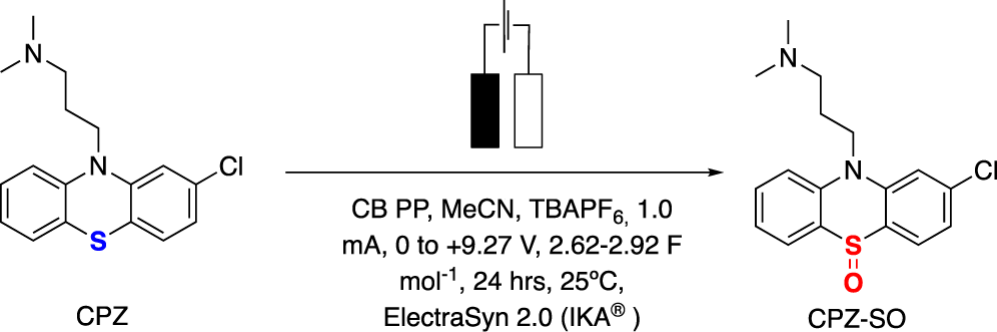
Reaction scheme for the conversion of CPZ to CPZ-sulfoxide.

Organic electrosynthesis is a field in the midst of a renaissance due to the ability to generate molecules through redox reactions in mild, safe, and green conditions while using electricity as the cheap and green electron source.^46^ Within electrosynthetic chemistry, it is common to utilize traditional aprotic solvents such as DMF and MeCN,^47^ both of which we now know that the 40 wt % CB/PP electrodes are stable in over at least a 15-day period. Due to the rapid prototyping capabilities of additive manufacturing, we designed and printed electrodes to fit the commercial ElectraSyn 2.0 and then applied them toward the electrosynthesis of chlorpromazine (CPZ) metabolites within MeCN. CPZ is a tranquilizing drug and antipsychotic used in the treatment of schizophrenia and related psychoses,^48,49^ with its main metabolites within humans being 7-hydroxy-CPZ, *N*-monodesmethyl-CPZ, and CPZ-sulfoxide.^50^ It has often been utilized as a one-electron mediator within electrochemistry and therefore its electrochemical behavior has been studied in the literature,^51−53^ including as a lyotropic liquid crystal.^54^ Its biological activity is thought to derive from its facile oxidation and photo-oxidation into a stable cation radical.^55^ In this work, we apply our additively manufactured electrodes produced from the 40 wt % CB/PP filament toward the synthesis of CPZ-sulfoxide within MeCN. A schematic overview of this reaction is explained in Figure 6.

In this, electrodes printed from the 25, 30, and 40 wt % CB/PP filament were iteratively applied to the electrosynthetic reaction and benchmarked to a commercially purchased glassy carbon electrode (GCE). Importantly, the material cost of making the additively manufactured electrodes was calculated to be only £0.06, in addition to the inherent customizable features offered by 3D-printing technology together with avoiding time-consuming and sometimes detrimental surface recovery pretreatments of classic solid electrodes.

Regarding the electrosynthesis of CPZ metabolites, a summary of the findings is included in Table 3, where the applied voltage, total charge, and importantly, the metabolite ratio are described. Additional information such as HPLC and NMR results can be found in Table S2 and Figures S5–19.

**Table 3:** Summary of the Electrosynthetic Parameters and Metabolite Ratios Achieved

				Metabolite Ratio	
Entry	Electrodes	Max Voltage Applied (V)	Total Charge (Fmol^–1^)	CPZ-sulfoxide	CPZ-sulfone
1	GCE	0 to +3.86	2.81	11	1
2	25% CB/PP	0 to +9.27	2.86	15	1
3	30% CB/PP	0 to +7.33	2.62	29	1
4	30% CB/PP*	0 to +6.00	2.66	35	1
5	40% CB/PP	0 to +4.91	2.88	10	1
6	40% CB/PP*	0 to +5.33	2.92	7	1

Our results showed that these electrodes functioned analogously to GCE with an improvement in the ratio between CPZ-sulfoxide and CPZ-sulfone (11:1 to 35:1). A favorable isolated yield of 68% of the desired mammalian CPZ-sulfoxide metabolite was achieved using the optimal 30% CB/PP (entry 3) for pharmaceutical evaluation. The yields in question were calculated as follows: %yield = (actual yield/theoretical yield) × 100. On the other hand, although they could be printed in bulk and disposed of after a single-use electrosynthesis experiment, in this work, we are looking into exploiting the chemical stability of this material to contribute to improving environmental sustainability in the application of 3D-printed CB/PP electrodes. As such, we reused the 30 and 40 wt % CB/PP electrodes in a second identical experiment to establish whether the electrodes were stable enough for multiple uses. In both cases, the results showed that the electrode could be reused, and for the 30 wt % CB/PP electrode, an improvement in the metabolite ratio was seen. This shows the excellent electrochemical performance and chemical stability of these materials and provides evidence of how they can unlock the field of nonaqueous electrochemistry for the additive manufacturing field.

## Conclusions

In this work, we report the first production and optimization of electrically conductive additive manufacturing filament from polypropylene (PP) with a carbon black (CB) loading of 40 wt % without additional plasticizer. The inclusion of CB provided enhanced thermal properties of the filament, while maintaining low-temperature flexibility for easy and high-quality printing. The electrodes printed from the filament were characterized through Raman spectroscopy, XPS, and SEM, as well as extensively electrochemically characterized. A *k*^0^ of 2.00 ± 0.04 × 10^–3^ cm s^–1^ was calculated for the 40 wt % CB/PP electrode, indicating this material has an electrochemical performance in line with the best PLA filaments reported in the literature.

The material was then applied toward three separate applications within electrochemistry, namely, aqueous electroanalysis, nonaqueous electrochemistry, and electrosynthesis. First, the electrodes were applied to the detection of CCH within environmental waters, where an LoD of 10 nM was obtained, which compared excellently with other reports in the literature and was 3× better than the only other additively manufactured electrode reported. The electrodes printed from the 40 wt % CB/PP were then shown to be exceptionally stable within organic solvents such as acetonitrile, dichloromethane, and dimethylformamide for up to 15 days, and for continuous 100 cyclic voltammograms, opening the field of new applications in nonaqueous electrochemistry. Finally, the electrodes were applied to the electrosynthesis of chlorpromazine metabolites within acetonitrile over a 24-h experiment. The conversion rate was shown to be vastly superior using the additively manufactured electrodes when compared to a conventional glassy carbon electrode, while having a material cost of only £0.06 per sensor. The production and successful printing of this material is paving the way for novel applications in additive manufacturing electrochemistry and has the potential to revolutionize the field.

## Experimental Section

### Chemicals

All chemicals used throughout this work were used as received without any further purification. All aqueous solutions were prepared with deionized water of a measured resistivity not less than 18.2 MΩ cm, sourced from a Milli-Q Integral 3 system from Millipore UK (Watford, UK). Hexaammineruthenium(III) chloride ([Ru(NH_3_)_6_]^3+^, 98%), potassium ferricyanide (99%), potassium ferrocyanide (98.5–102%), sodium hydroxide (>98%), potassium chloride (99.0–100.5%), chlorpromazine (CPZ, ≥98%), tetrabutylammonium hexafluorophosphate (TBAPF_6_, ≥99.0%), boric acid (≥99.5%), sodium tetraborate (99%), sodium chloride (≥99.0%), acetic acid (≥99%), phosphoric acid (≥85%), and phosphate-buffered saline (PBS) tablets were purchased from Merck (Gillingham, UK). Acetonitrile (99.9%), dichloromethane (99.8%), and *N*,*N*-dimethylformamide (99.8%) were purchased from Fisher Scientific (Loughborough, UK). CCH (≥97.0%) was purchased from TCI Europe (Zwijndrecht, Belgium). Carbon black (CB) was purchased from PI-KEM (Tamworth, UK). Poly(propylene) (PP, Sabic CX03–81 Natural 00900) was purchased from Hardie Polymers (Glasgow, UK). Commercial conductive CB/PLA filament (1.75 mm, ProtoPasta, Vancouver, BC, Canada) was purchased from Farnell (Leeds, UK). River water was supplied and collected by the Uberlândia Water and Sewer Department (Minas Gerais, Brazil) at the Bom Jardim Treatment Plant from the Bom Jardim stream, a tributary of the Uberabinha River (approximate location: 18.943144° S 48.272647° W). Collection procedures were performed by the National Guideline for the Sampling Plan of the Surveillance of Water Quality for Human Consumption of the Brazilian Ministry of Health. Tap water samples were obtained from laboratory 5.39, John Dalton Tower, Manchester, UK. Bottled water samples were Highland Spring Still Water (500 mL) obtained from a local convenience store.

### Filament Production

The polymer compositions were prepared through the addition of appropriate amounts of PP and CB in a chamber of 63 cm^3^. The compounds were mixed using a Thermo Haake Polydrive dynameter fitted with a Thermo Haake Rheomix 600 (Thermo-Haake, Germany) at 210 °C with Banbury rotors at 70 rpm for 5 min. The resulting polymer composites were allowed to cool to room temperature before being granulated to create a finer particle size by using a Rapid Granulator 1528 (Rapid, Sweden). The polymer composites were collected and processed through the hopper of an EX2 extrusion line (Filabot, VA, U.S.A.). The EX2 was set up with a single screw with a set heat zone of 210 °C. The molten polymer was extruded from a 1.75 mm die head, pulled along an Airpath cooling line (Filabot, VA, U.S.A.) and collected on a spool. After this, the filament was ready to use for Additive Manufacturing (AM).

### Additive Manufacturing of the Electrodes

All computer designs and 3MF files in this manuscript were produced using Fusion 360 (Autodesk, CA, U.S.A.). These files were sliced and converted to GCODE files in PrusaSlicer (Prusa Research, Prague, Czech Republic). The additive-manufactured electrodes were produced using fused filament fabrication (FFF) technology on a Prusa i3MK3S+ (Prusa Research, Prague, Czech Republic). All additive-manufactured electrodes were printed using identical printing parameters, namely a 0.6 mm nozzle with a nozzle temperature of 245 °C, 100% rectilinear infill,^7^ 0.15 mm layer height, print speed of 35 mm s^–1^, and bed temperature of 110 °C. For better adhesion of the PP to the surface of the printing bed, one layer of glue (Magigoo—the 3D printing adhesive single pen) was applied to the printing area 10 min before heating the bed.

### Physicochemical Characterization

Thermogravimetric analysis (TGA) was performed using a Discovery Series SDT 650 instrument controlled by Trios Software (TA Instruments, DE, USA). Samples were mounted in alumina pans (90 μL) and tested using a ramp profile (10 °C min^–1^) from 0 to 800 °C under N_2_ (100 mL min^–1^).

X-ray Photoelectron Spectroscopy (XPS) data were acquired using an AXIS Supra (Kratos, UK), equipped with a monochromatic Al X-ray source (1486.6 eV) operating at 225 W and a hemispherical sector analyzer. It was operated in fixed transmission mode with a pass energy of 160 eV for survey scans and 20 eV for region scans with the collimator operating in slot mode for an analysis area of approximately 700 × 300 μm; the fwhm of the Ag 3d5/2 peak using a pass energy of 20 eV was 0.613 eV. The binding energy scale was calibrated by setting the graphitic sp^2^ C 1s peak to 284.5 eV; this calibration is acknowledged to be flawed^56^ but was nonetheless used in the absence of reasonable alternatives, and because only limited information was to be inferred from absolute peak positions.

Scanning Electron Microscopy (SEM) micrographs were obtained using a Crossbeam 350 Focused Ion Beam—Scanning Electron Microscope (FIB-SEM) (Carl Zeiss Ltd., Cambridge, UK) fitted with a field emission electron gun. Secondary electron imaging was completed using a secondary electron and secondary ion (SESI) detector. Samples were mounted on the aluminum SEM pin stubs (12 mm diameter, Agar Scientific, Essex, UK) using adhesive carbon tabs (12 mm diameter, Agar Scientific, Essex, UK) and coated with a 5 nm layer of Au/Pd metal using a Leica EM ACE200 coating system before imaging.

Raman spectroscopy was performed on a DXR Raman Microscope (Thermo Scientific Inc., Waltham, MA, U.S.A.) configured with a 532 nm laser and operated using OMNIC 9 software.

### Electrochemical Experiments

All electrochemical experiments were performed on an Autolab 100N potentiostat controlled by NOVA 2.1.7 (Utrecht, The Netherlands). Identical additive manufactured electrodes were used throughout this work for all filaments, printed in a lollipop shape (Ø 5 mm disc with 8 mm connection length and 2 × 1 mm thickness^57^) alongside an external commercial Ag|AgCl/KCl (3M) reference electrode with a nichrome wire counter electrode. All solutions of [Ru(NH_3_)_6_]^3+^ were purged of the O_2_ thoroughly using N_2_ prior to any electrochemical experiments. Solutions of [Fe(CN)_6_]^4-/3–^ were prepared in the same way without the need for further degassing.

Electrochemical impedance spectroscopy (EIS) was recorded in the frequency range of 0.1 Hz to 100 kHz applying 10 mV of signal amplitude to perturb the system under quiescent conditions. NOVA 2.1.7 software was used to fit Nyquist plots obtained to an adequate equivalent circuit.

Activation of the additive manufactured electrodes was performed before all electrochemical experiments. This was achieved electrochemically in NaOH (0.5 M), as described in the literature.^58^ Briefly, the additive manufactured electrodes were connected as the working electrode in conjunction with a nichrome wire coil counter and an Ag|AgCl/KCl (3 M) reference electrode and placed in a solution of 0.5 M NaOH. Chronoamperometry was used to activate the additive-manufactured electrodes by applying a set voltage of +1.4 V for 200 s, followed by applying −1.0 V for 200 s. The additive manufactured electrodes were then thoroughly rinsed with deionized water and dried under compressed air before further use.

The HET rate constants, , were calculated as an average of 3 sets of 10 different scan rates (5, 10, 15, 25, 50, 75, 100, 150, 250, and 500 mV s^–1^), where each set used a new AME. These were performed using the near ideal outer-sphere redox probe RuHex (in 0.1 M KCl) using the well-known^59^ and widely utilized Nicholson method,^60^ for *quasi*-reversible electrochemical reactions via the following formula:^61^
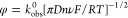
1where φ is a kinetic parameter, *D* is the diffusion coefficient for RuHex (*D* = 9.1 × 10^–6^ cm^2^ s^–1^),^59^*n* is the number of electrons that are taking part in the process, *F* is the Faraday constant, *v* is the scan rate, *R* is the gas constant, and *T* is the temperature in Kelvin. In order to calculate the HET rate constant, we use the peak-to-peak separation (Δ*E*_p_) to deduce *φ,* where Δ*E*_p_ is obtained at various voltammetric scan rates.^62^ The standard heterogeneous constant () can be calculated via the gradient when plotting φ against [*πDnvF*/*RT*]^−1/2^. In cases where Δ*E*_p_ is bigger than 212 mV, the following equation should be implemented:
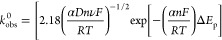
2where α is assumed to be 0.5.^63,64^

The electroactive area of the electrode, *A*_real_, is calculated using the Randles-Ševćik equation at nonstandard conditions for quasi- (3) and irreversible (4) electrochemical processes when appropriate:^65^
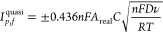
3
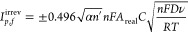
4where in all cases, *n* is the number of electrons in the electrochemical reaction, *I*_p,f_ is the voltammetric current (analytical signal) using the first peak of the electrochemical process, *F* is the Faraday constant (C mol^–1^), *v* is the applied voltammetric scan rate (V s^–1^), *R* is the universal gas constant, *T* is the temperature in Kelvin, *A*_real_ is the electroactive area of the electrode (cm^2^), *D* is the diffusion coefficient (cm^2^ s^–1^), and α is the transfer coefficient (usually assumed to be close to 0.5). Following the calculation of *A*_real_, the percentage of the geometrical area was calculated using the following formula: *%*Real_Area_= (*A*_real_/*A*_geo_) × 100. Limits of detection (LoD) were calculated as 3 times the standard deviation of the blank (3σ) divided by the slope of the calibration plot.

For studies using CCH, a stock solution (1.0 mM) was prepared by dissolving CCH directly in the supporting electrolyte, and the necessary dilutions were performed for subsequent studies. Both bottled and tap water samples were diluted (20-folds) in the supporting electrolyte to perform the analyses. However, the river water sample was not diluted; in this case, the supporting electrolyte was prepared using river water as a solvent. No additional preparation step was used.

### Electrosynthesis

Reactions were monitored by TLC analysis on Merck silica gel 60 F254 using UV light (254 nm) or potassium permanganate. ^1^H and ^13^C NMR spectra were recorded either on a Bruker AVIII operating at 300 MHz for ^1^H and fitted with a 5 mm BBFO probe or on a Bruker AVANCE NEO operating at 400 MHz for ^1^H fitted with a 5 mm “smart” BBFO probe, respectively. Chemical shift data for ^1^H are reported in parts per million (ppm, δ scale) downfield from tetramethylsilane (TMS: δ 0.0) and referenced internally to the residual proton in the solvent. The deuterated solvents used for NMR analysis were chloroform (CDCl_3_: δH 7.26, δC 77.2), methanol (MeOD: δH 3.31, δC 49.2), and dimethyl sulfoxide (DMSO-d6: δH 2.50, δC 39.5). Coupling constants (*J*) are given in hertz (Hz). The data are presented as follows: chemical shift, multiplicity (s = singlet, d = doublet, t = triplet, q = quartet, p = pentet, m = multiple, br = broad, app = apparent, and combinations thereof), coupling constant, and integration and assignment. Mass spectra were recorded on a Waters Xevo G2-XS ToF or Synap G2-S mass spectrometer using Zspray and electrospray ionization in negative (ESI−) and positive (ESI+) mode, respectively.

### Samples and Solutions Preparation

Chlorpromazine (CPZ) (100 mg, 0.31 mmol) and tetrabutylammonium hexafluorophosphate (TBAPF_6_) (608 mg, 1.57 mmol) (analyte: electrolyte 1:5) were dissolved in acetonitrile (MeCN) (12.0 mL).

### Electrosynthesis of Chlorpromazine Metabolites

Electrosynthesis of CPZ metabolites was performed using ElectraSyn 2.0 (IKA) under a constant current control. An undivided glass cell (electrochemical vial) equipped with a magnetic stirrer was added to the analyte solution under study. Glassy carbon electrodes (GCE) (IKA, dimensions (*W* × *H* × *D* = 8 × 52.5 × 2 mm) as the control standard electrodes or iterative variations of 25%, 30%, and 40% CB PP electrodes (dimensions (*W* × *H* × *D* = 8 × 52.5 × 2 mm) as both the working electrode (WE) and the counter electrode (CE) were inserted into the solution at an opposing distance of ∼5 mm. Prior to the experiment, the electrodes were rinsed with double-distilled, deionized water, followed by MeCN, and allowed to air-dry. A fixed current (1.0 mA) was passed through the solution for 24 h with a stirring speed of 500 rpm. The electrolysis product was analyzed and monitored using TLC (SiO_2_, eluent – DCM: methanol– 90:10), and the TBAPF_6_ was removed by recrystallization before HPLC analysis.

### Recrystallization of TBAPF_6_

The reaction mixture in MeCN was decanted into the flask, and MeCN was evaporated using a rotary evaporator. Methanol (15 mL) was added to dissolve the crude product and evaporated until crystals of TBAPF_6_ formed. The crystals of TBAPF_6_ were separated from the filtrate, and the filtrate was cooled overnight in the fridge (0 °C), resulting in a secondary batch of TBAPF_6_ crystals formed. The crystals of TBAPF_6_ were collected either by filtration or using a Pasteur pipet to collect and separate the filtrate containing a mixture of chlorpromazine metabolites.

### Metabolite Purification

The purification of chlorpromazine metabolites was performed using the Biotage Isolera system as follows: Biotage Sfär silica high-capacity duo columns 20 μm in diameter (25 g) were obtained with Samplet. Mobile phase: [A] DCM; [B] Methanol. Gradient: 0% B for 5 CV length to 10% B for 10 CV length; held for 15 CV. Flow rate: 35 mL/min. UV detectors: 254 and 280 nm.

### HPLC Methodology

HPLC: Thermo Scientific Vanquish Flex UHPLC. Ascentis C_18_ HPLC Column RP-Amide, 25 cm × 4.6 mm I.D., 5 μm particles (581325-U). Mobile phase: [A] 0.05% TFA in water; [B] acetonitrile (3:7). Flow rate: 1.0 mL/min. Detector: UV 254 nm. Injection: 5 μL. Sample: 3 mg of sample in 20 mL of acetonitrile (0.15 mg/mL).

## Supporting Information Available

Table of filament and electrode resistances, photographs of filament, scan rate studies, the proposed mechanism for CCH, buffer analytical calibration plot for CCH, optimization of HPLC protocol, and HPLC data for the electrosynthetic reactions (PDF)
