# Supplementary material for: Conductive Polypropylene Additive Manufacturing Feedstock: Application to Aqueous Electroanalysis and Unlocking Nonaqueous Electrochemistry and Electrosynthesis
Source: ACS Appl Mater Interfaces. 2024 Oct 2;16(41):56006–18. doi: 10.1021/acsami.4c12967 (PMC11492246; doi:10.1021/acsami.4c12967)
Supplement: Supplementary file 1 — am4c12967_si_001.pdf [file am4c12967_si_001.pdf]

Supporting Information for:

Conductive polypropylene additive manufacturing feedstock: application to aqueous electroanalysis and unlocking non-aqueous electrochemistry and electrosynthesis

David L. O. Ramos,[1,2] Robert D. Crapnell,[1] Ridho Asra,[3] Elena Bernalte,[1] Ana Oliveira,[1,2] Rodrigo A. A. Muñoz,[2] Eduardo M. Richter,[2] Alan M. Jones[3*]

and Craig E. Banks[1*]

[1] Faculty of Science and Engineering, Manchester Metropolitan University, Dalton Building, Chester Street, M1 5GD, Great Britain.

[2] Institute of Chemistry, Federal University of Uberlândia, 38400-902 Uberlândia, Minas Gerais, Brazil.

[3] School of Pharmacy, University of Birmingham, Edgbaston, Birmingham B15 2TT, United Kingdom.

*To whom correspondence should be addressed.

E-mail: c.banks@mmu.ac.uk; Tel: +44(0)1612471196

Email: Alan Jones; a.m.jones.2@bham.ac.uk

Table S1. Bulk resistances measured for each composition of CB in PP along 10 cm of filament and along the connection length of the lollipop electrodes. (n = 6).

Filament Composition	Filament Resistance (Ω)	Electrode Resistance (Ω)
15% CB	32,000 ± 7,000	17,000 ± 3,000
20% CB	7,000 ± 500	2,000 ± 200
25% CB	2,000 ± 100	388 ± 70
30% CB	762 ± 52	161 ± 40
35% CB	422 ± 39	92 ± 6
40% CB	247 ± 30	55 ± 4

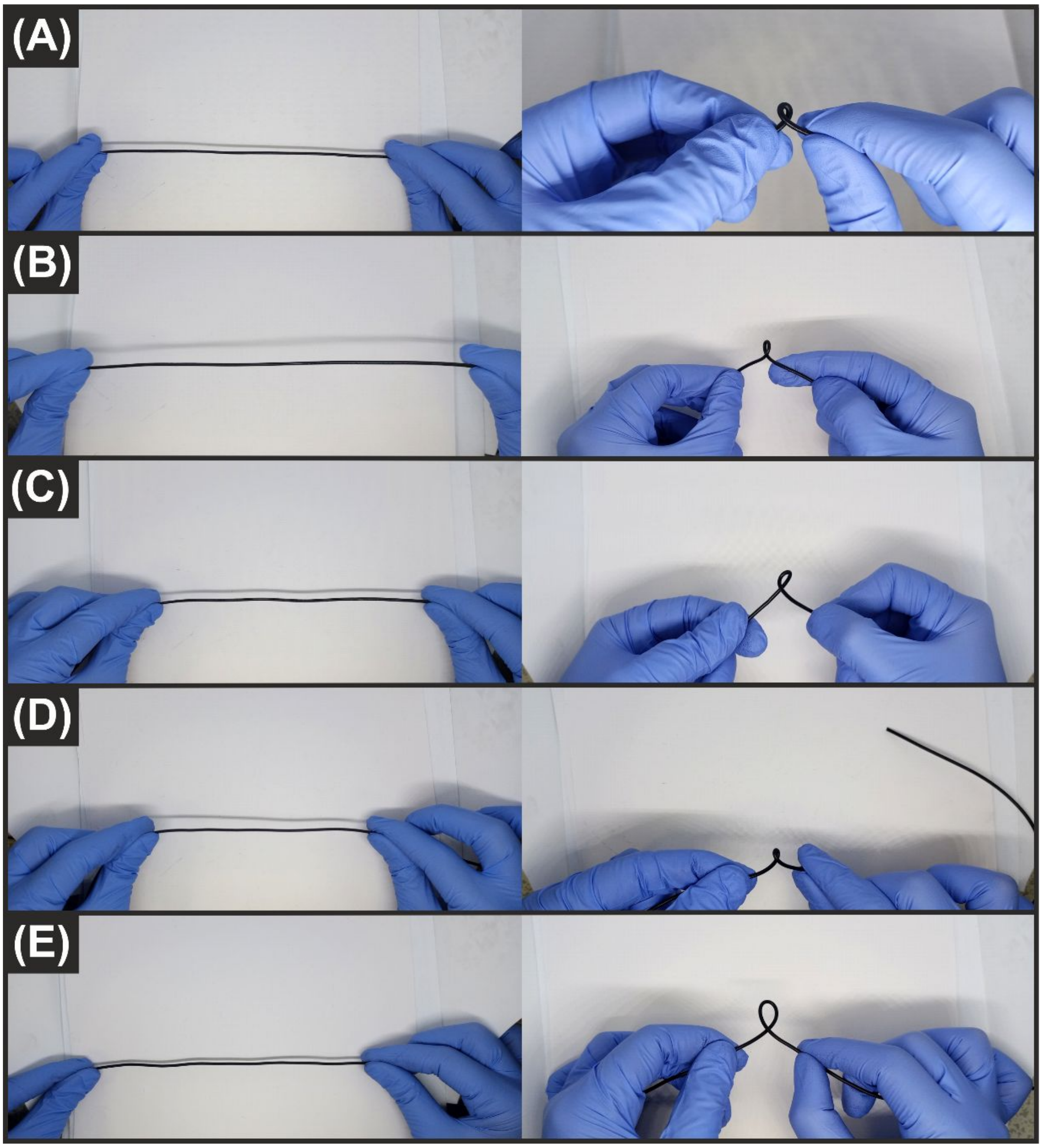

Figure S1. Photographs of the different bespoke filaments straightened and bent to exhibit the excellent low-temperature flexibility. **A)** 15 wt% CB; **B)** 20 wt% CB; **C)** 25 wt% CB; **D)** 30 wt% CB; **E)** 35 wt% CB.

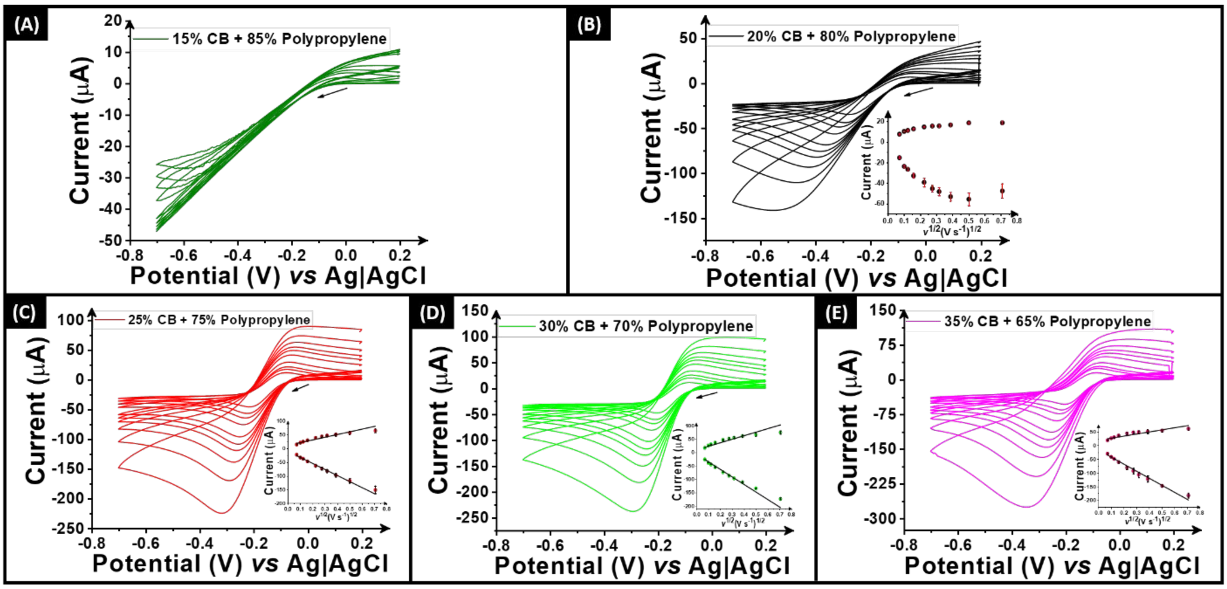

Figure S2. Scan rate study (5 – 500 mV s^{-1}) with $[Ru(NH_3)_6]^{3+}$ (1 mM in 0.1 M KCl) performed in the **A)** 15% CB + 85% PP, **B)** 20% CB + 80% PP, **C)** 25% CB + 75% PP, **D)** 30% CB + 70% PP, and **E)** 35% CB + 65% PP as the WE. Inset: the Randles–Ševčík plot.

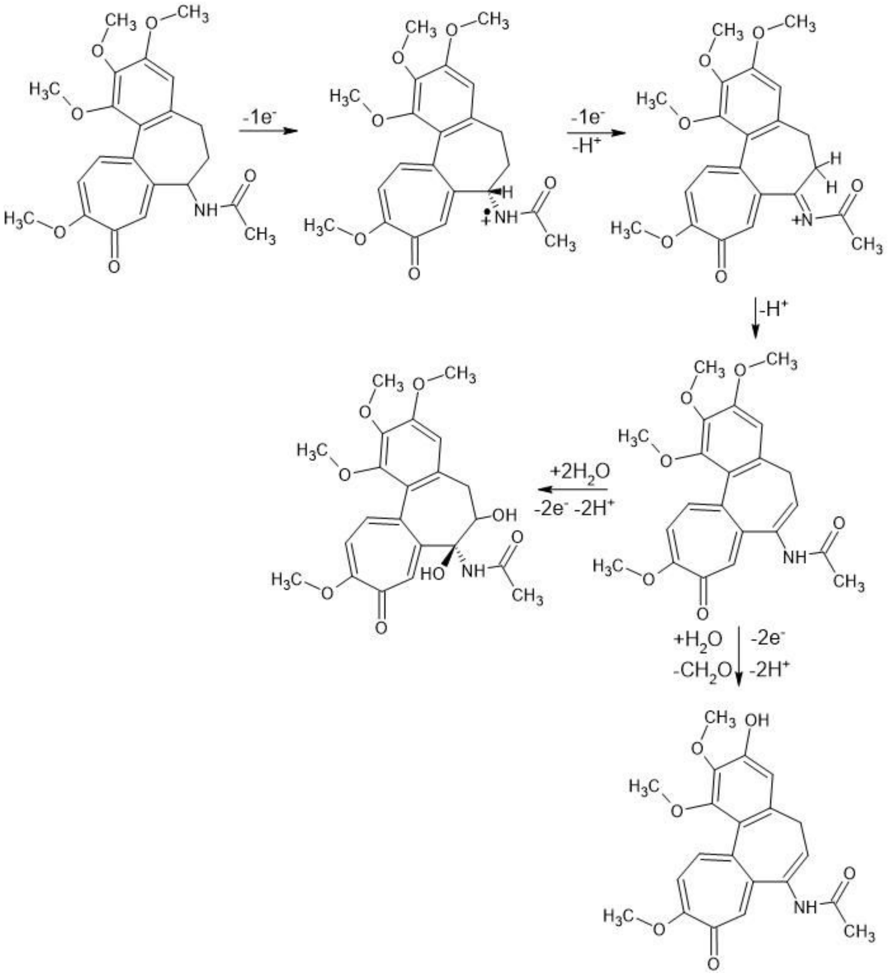

Figure S3. Proposed mechanism for CCH[1]

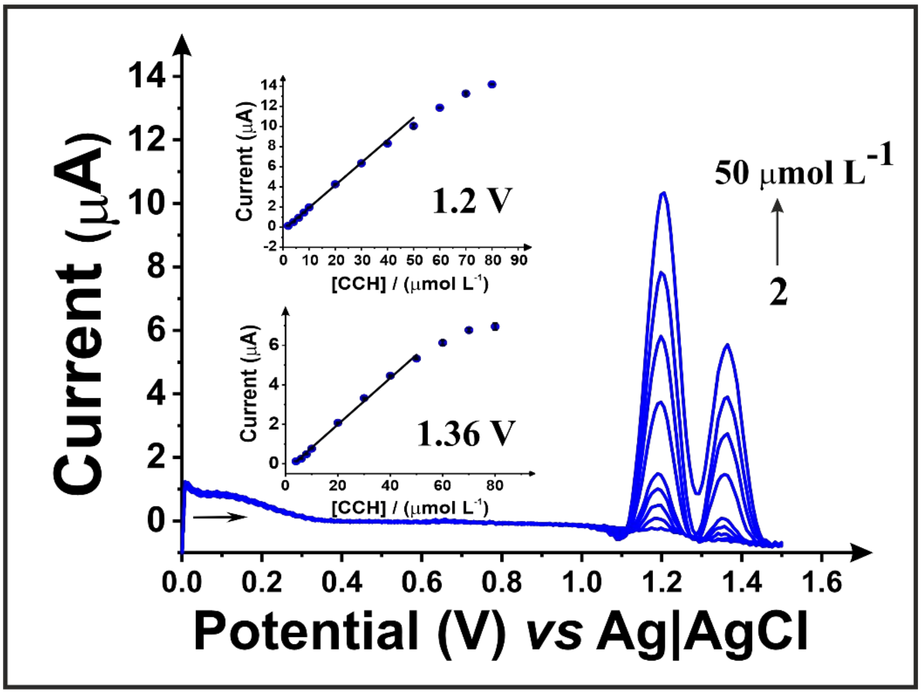

Figure S4. SW voltammograms for CCH oxidation performed at 40 wt% CB in PP electrode in 0.1 mol L^{-1} borate buffer pH 9 in the concentration range of 2 to 50 μmol L^{-1}. Inset: calibration plots for the two oxidation peaks of CCH: +1.20 (I (μA) = -0.300 ± 0.042 [CCH] (μmol L^{-1} μA^{-1}) + 0.220 ± 0.003, R^2 = 0.998) and +1.36 V (I (μA) = -0.360 ± 0.003 [CCH] (μmol L^{-1} μA^{-1} + 0.120 ± 0.003), R^2 = 0.996).

Table S2. Gradient Program on Chromaleon 7 Thermo Scientific: **(A): Water 0.05% TFA (B) Acetonitrile**

No	Time	Flow (mL/min)	%A	%B	Curve
1	0	Equilibration			
2	0	1	90	10	5
3		Run			
4	1	1	90	10	5
5	16	1	15	85	5
6	18	1	15	85	5
7	19	1	90	10	5
8	21.5	1	90	10	5
9	25	Stop Run			

Figure S5. *Chlorpromazine (CPZ)* (3 mg/20 mL acetonitrile; 0.15 mg/mL)

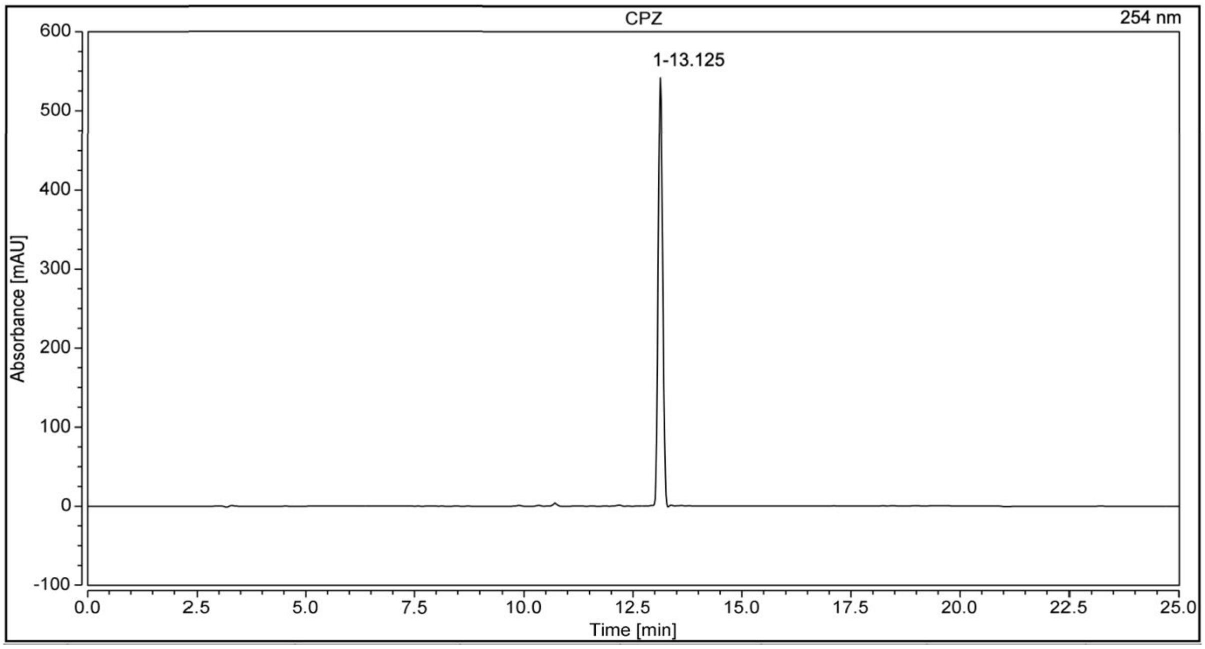

No.	Peak Name	Retention Time	Area mAU*min	Height mAU	Relative Area %	Relative Height %
1	Chlorpromazine	13.125	65.694	542.568	76.15	94.34

Figure S6. *CPZ-sulfoxide metabolite*

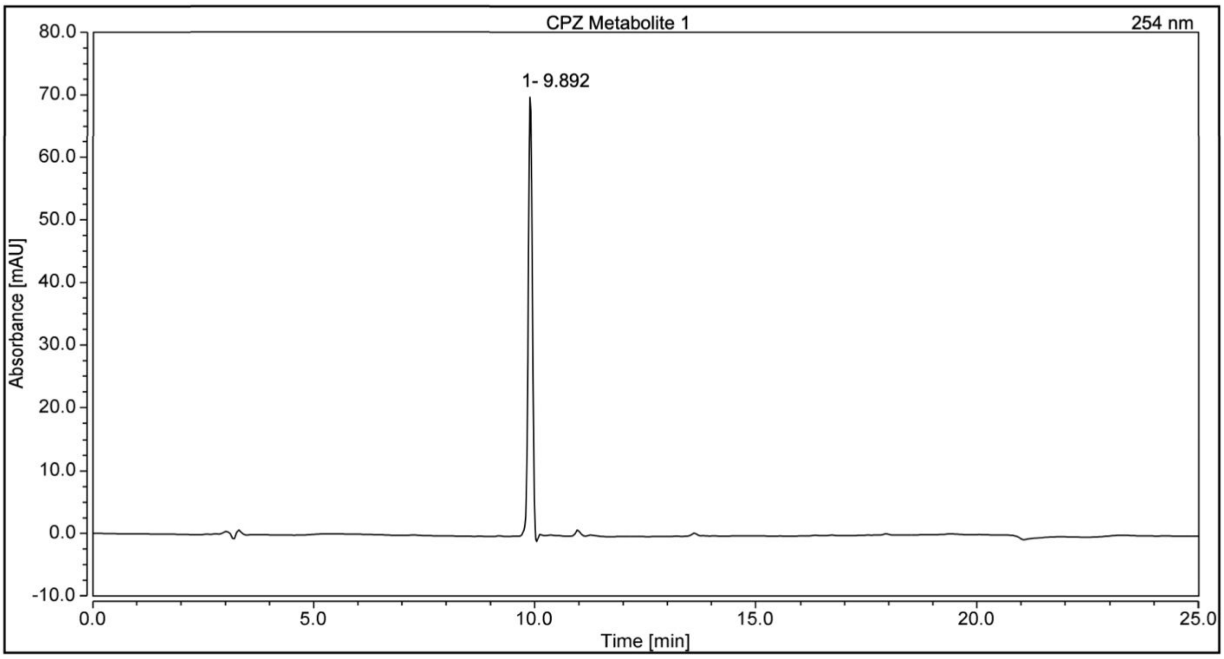

No.	Peak Name	Retention Time	Area mAU*min	Height mAU	Relative Area %	Relative Height %
1	CPZ-sulfoxide	9.892	7.747	70.943	42.17	87.65

Figure S7. *CPZ-sulfone metabolite*

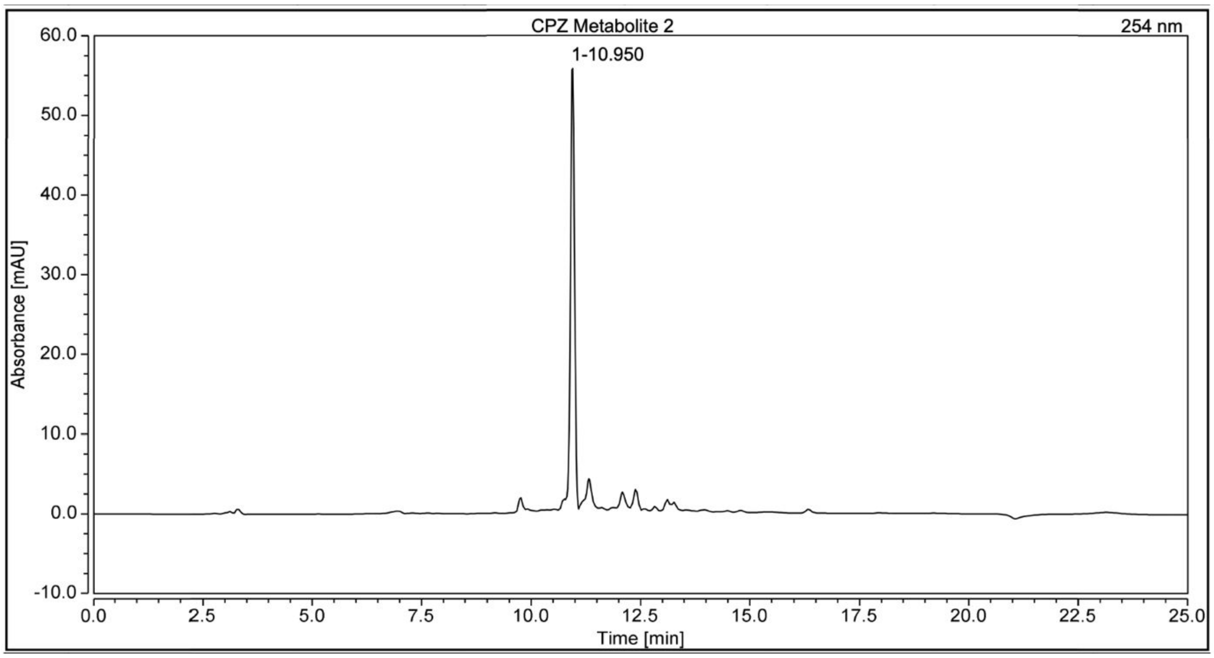

No.	Peak Name	Retention Time	Area mAU*min	Height mAU	Relative Area %	Relative Height %
1	CPZ-sulfone	10.950	6.217	55.975	38.03	60.72

Figure S8. *Entry 1: Glassy Carbon Electrode (GCE-IKA) (1.0 mA, 24 hrs, 2.81 F/mol, Maximum applied voltage 3.86 V)*

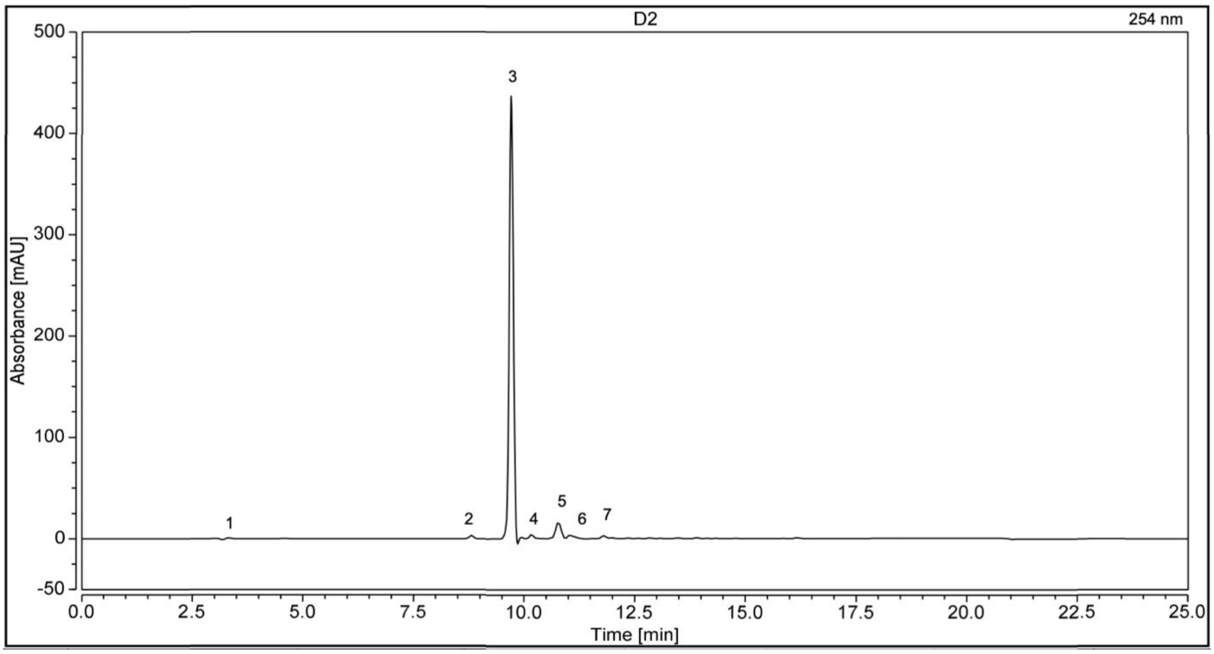

No.	Rt	Name	AUC
1	3.308	Unknown	1.818
2	8.808	Unknown	4.430
3	9.808	**CPZ-sulfoxide**	50.30
4	10.175	Unknown	2.698
5	10.975	**CPZ-sulfone**	4.506
6	11.142	Unknown	1.762
7	11.792	Unknown	1.813

Figure S9. *Entry 2: 25% CB PP (1.0 mA, 24 hrs, 2.86 F/mol, Maximum applied voltage 9.27 V)*

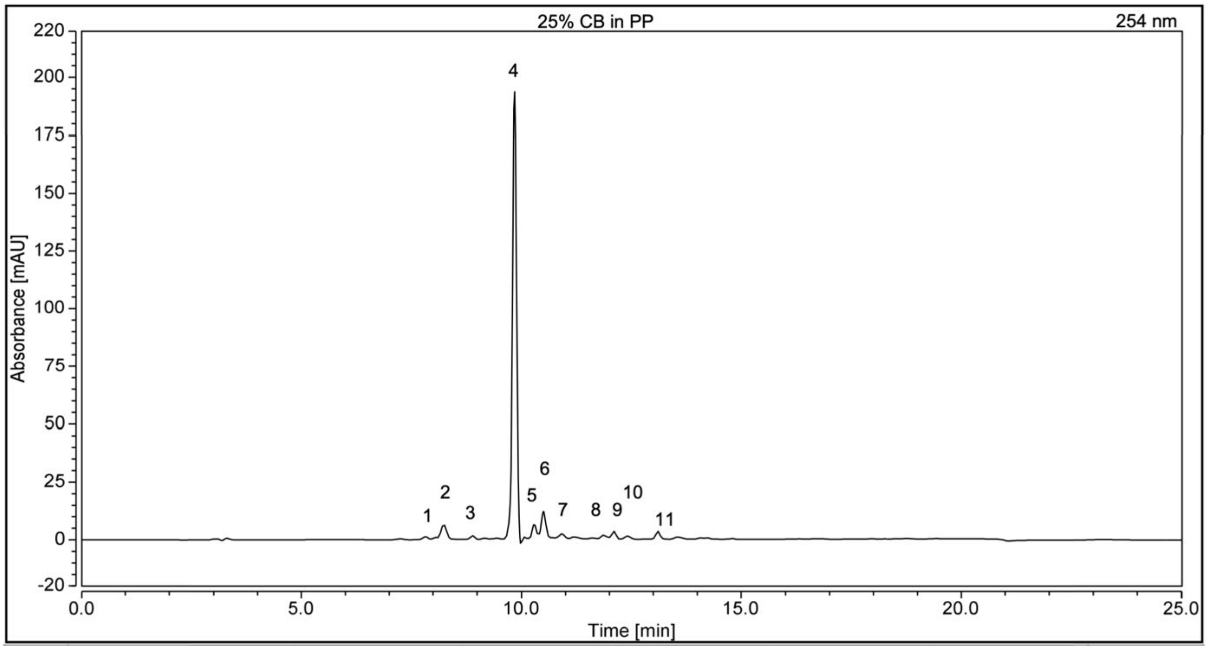

No.	Rt	Name	AUC
1	7.825	Unknown	0.727
2	8.242	Unknown	1.688
3	8.908	Unknown	0.843
4	9.842	**CPZ-sulfoxide**	21.689
5	10.292	Unknown	1.294
6	10.492	Unknown	2.141
7	10.908	**CPZ-sulfone**	1.475
8	11.858	Unknown	0.971
9	12.108	Unknown	1.104
10	12.842	Chlorpromazine	0.274
11	13.108	**CPZ**	1.111

Figure S10. *Entry 3: 30% CB PP (1.0 mA, 24 hrs, 2.62 F/mol, Maximum applied voltage 7.33 V)*

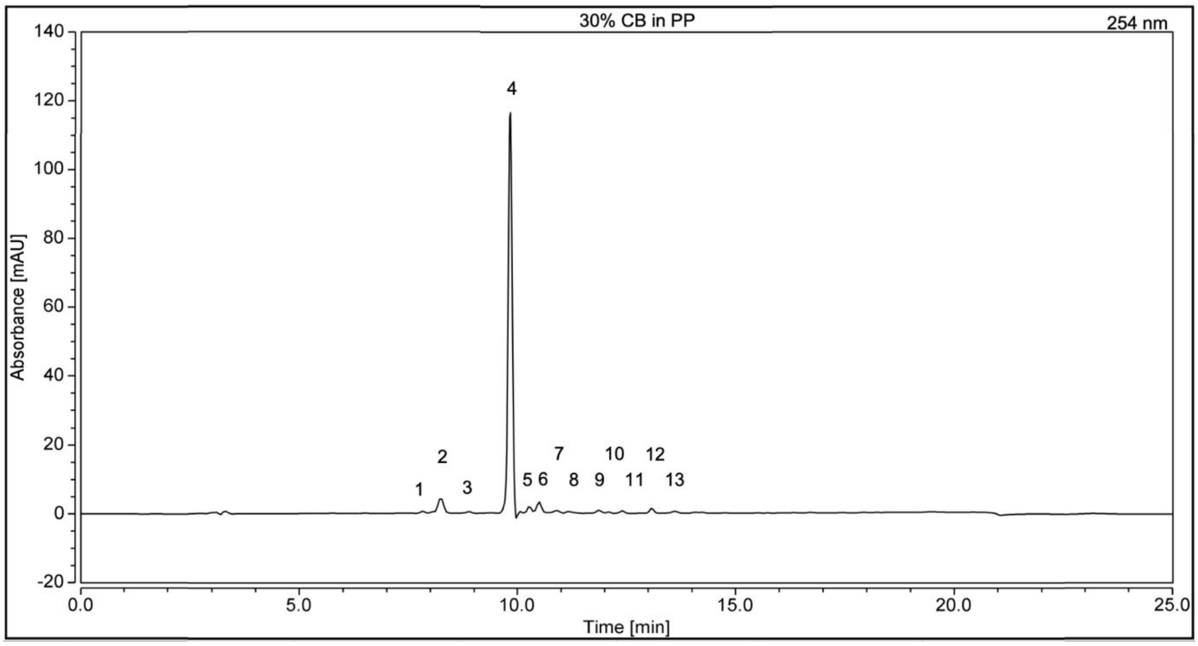

No.	Rt	Name	AUC
1	7.825	Unknown	0.104
2	8.242	Unknown	3.021
3	8.875	Unknown	0.162
4	9.842	**CPZ-sulfoxide**	13.044
5	10.275	Unknown	0.501
6	10.492	Unknown	0.836
7	10.892	**CPZ-sulfone**	0.450
8	11.158	Unknown	0.537
9	11.858	Unknown	0.378
10	12.092	Unknown	0.029
11	12.408	Unknown	0.094
12	13.075	**CPZ**	0.160
13	13.608	Unknown	0.101

Figure S11. *Entry 4: 30% CB PP (1.0 mA, 24 hrs, 2.66 F/mol, Maximum applied voltage 6.00 V)*

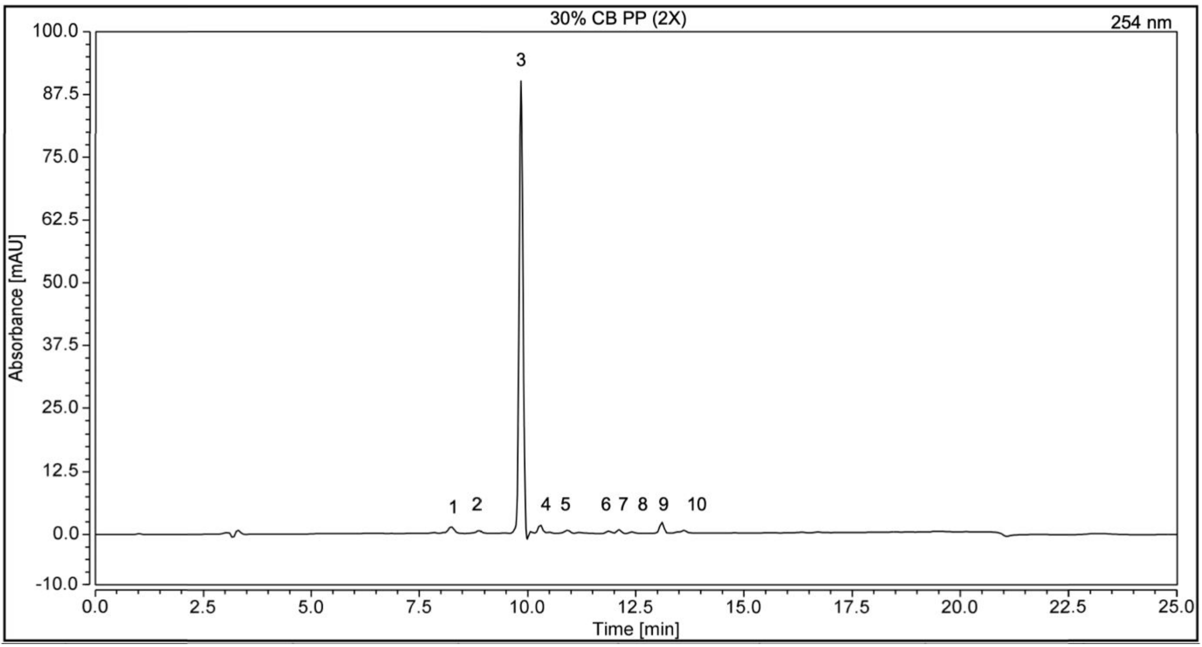

No.	Rt	Name	AUC
1	8.242	Unknown	0.269
2	8.875	Unknown	0.102
3	9.842	**CPZ-sulfoxide**	9.762
4	10.492	Unknown	0.306
5	10.908	**CPZ-sulfone**	0.283
6	11.858	Unknown	0.198
7	12.108	Unknown	0.250
8	12.408	Unknown	0.098
9	13.092	**CPZ**	0.246
10	13.608	Unknown	0.104

Figure S12. *Entry 5: 40% CB PP (1.0 mA, 24 hrs, 2.88 F/mol, Maximum applied voltage 4.91 V*

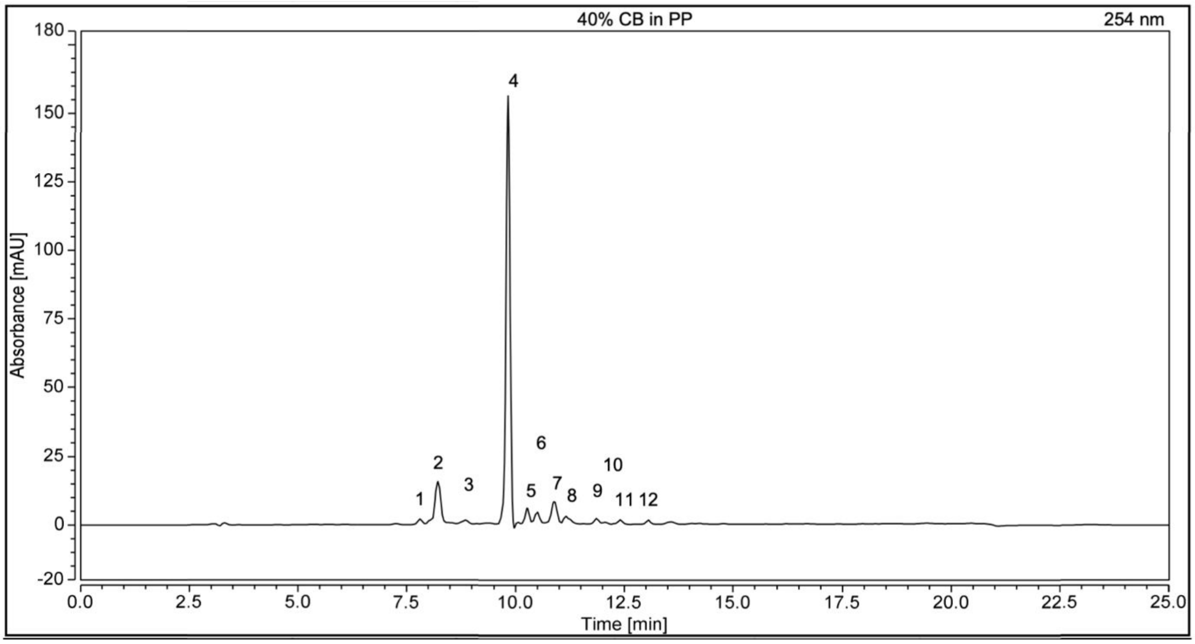

No.	Rt	Name	AUC
1	7.808	Unknown	0.625
2	8.225	Unknown	2.712
3	8.858	Unknown	0.429
4	9.825	**CPZ-sulfoxide**	17.344
5	10.258	Unknown	0.932
6	10.492	Unknown	0.889
7	10.892	**CPZ-sulfone**	1.670
8	11.258	Unknown	0.436
9	11.858	Unknown	0.576
10	12.075	Unknown	0.044
11	12.408	Unknown	0.218
12	13.042	**CPZ**	0.166

Figure S13. *Entry 6: 40% CB PP (1.0 mA, 24 hrs, 2.92 F/mol, Maximum applied voltage 5.33 V*

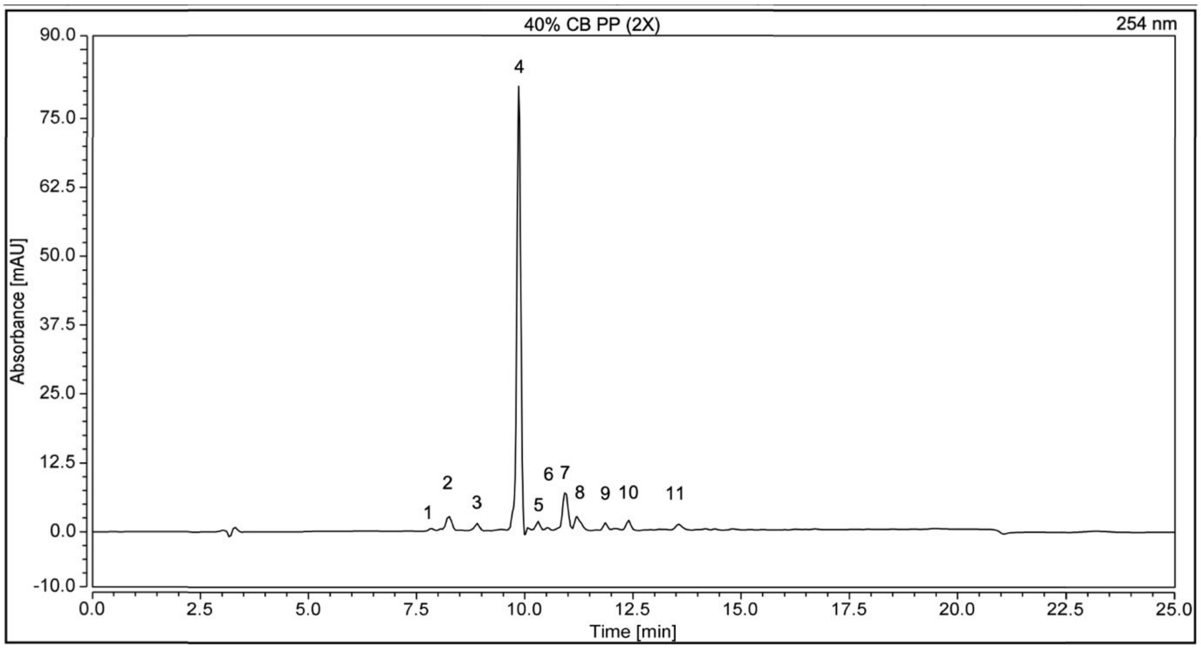

No.	Rt	Name	AUC
1	7.842	Unknown	0.051
2	8.242	Unknown	0.416
3	8.908	Unknown	0.172
4	9.858	**CPZ-sulfoxide**	8.861
5	10.308	Unknown	0.388
6	10.525	Unknown	0.081
7	10.942	**CPZ-sulfone**	1.326
8	11.208	Unknown	0.647
9	11.858	Unknown	0.412
10	12.392	Unknown	0.373
11	13.558	Unknown	0.520

Compound Characterisation

CPZ-sulfoxide

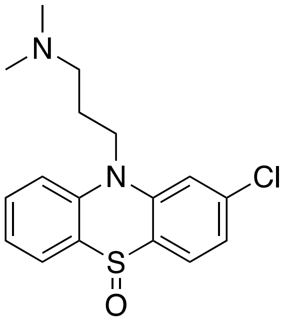

Following the general electrosynthesis procedure CPZ (100 mg, 0.31 mmol), TBAPF6 (608 mg, 1.57 mmol), and the work-up procedures afforded the title compound (71 mg, 0.21 mmol, 68%) as a brown solid. **M.P** 157-161°C. **^{1}H NMR** (400 MHz, DMSO-d_6) δ_H 8.13 – 7.16 (m, 7H), 4.41 (t, J = 7.4 Hz, 2H), 2.70 (td, J = 7.1, 3.0 Hz, 2H), 2.38 (s, 6H), 2.09 – 1.92 (m, 2H). **^{13}C NMR** (101 MHz, DMSO) δ_C 139.1, 137.7, 137.4, 133.2, 132.8, 131.1, 124.6, 123.2, 122.4, 121.7, 116.87, 116.3, 54.7, 44.5, 43.8, 22.7. **LRMS** (ES^+) *m/z* 335.10 ($[M+H]^+$, 100%). **HRMS** (EI^+) *m/z* $C_{17}H_{19}ClN_2OS$ requires 334.0901, found 334.0904 ($[M]^+$). Data is in accordance with literature. [2]

Figure S14. **^{1}H NMR** (400 MHz, DMSO-d_6) of **CPZ-sulfoxide**

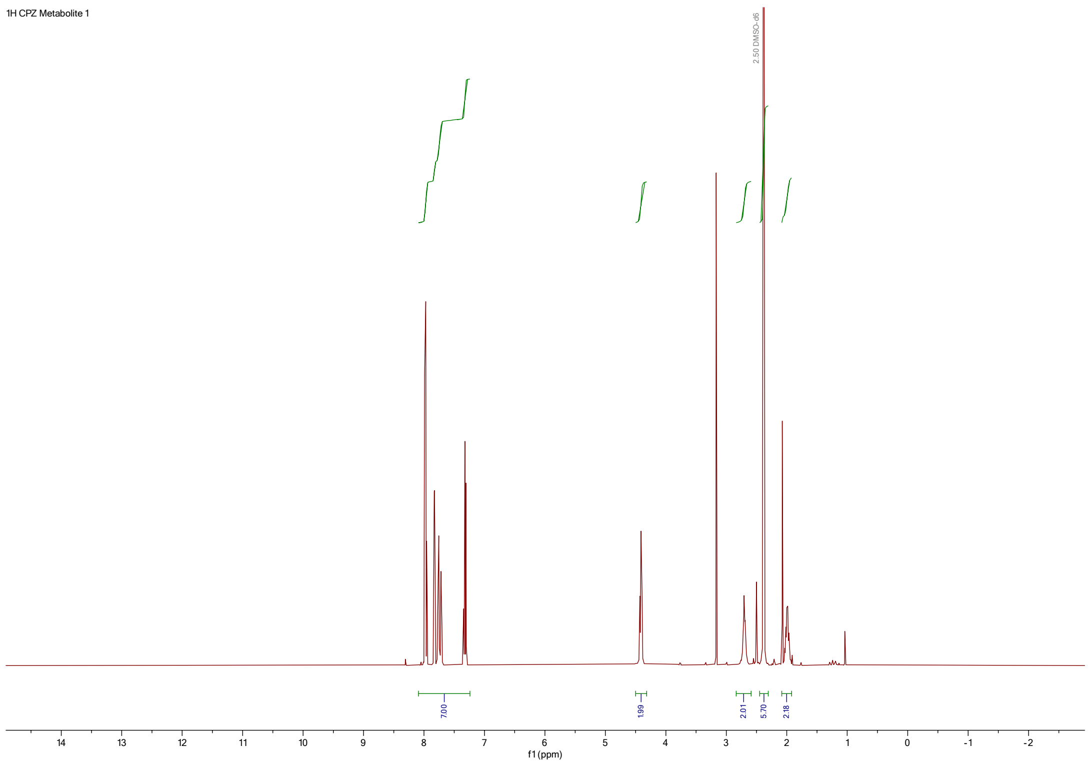

Figure S15. **^{13}C NMR** (101 MHz, DMSO-d_6) of **CPZ-sulfoxide**

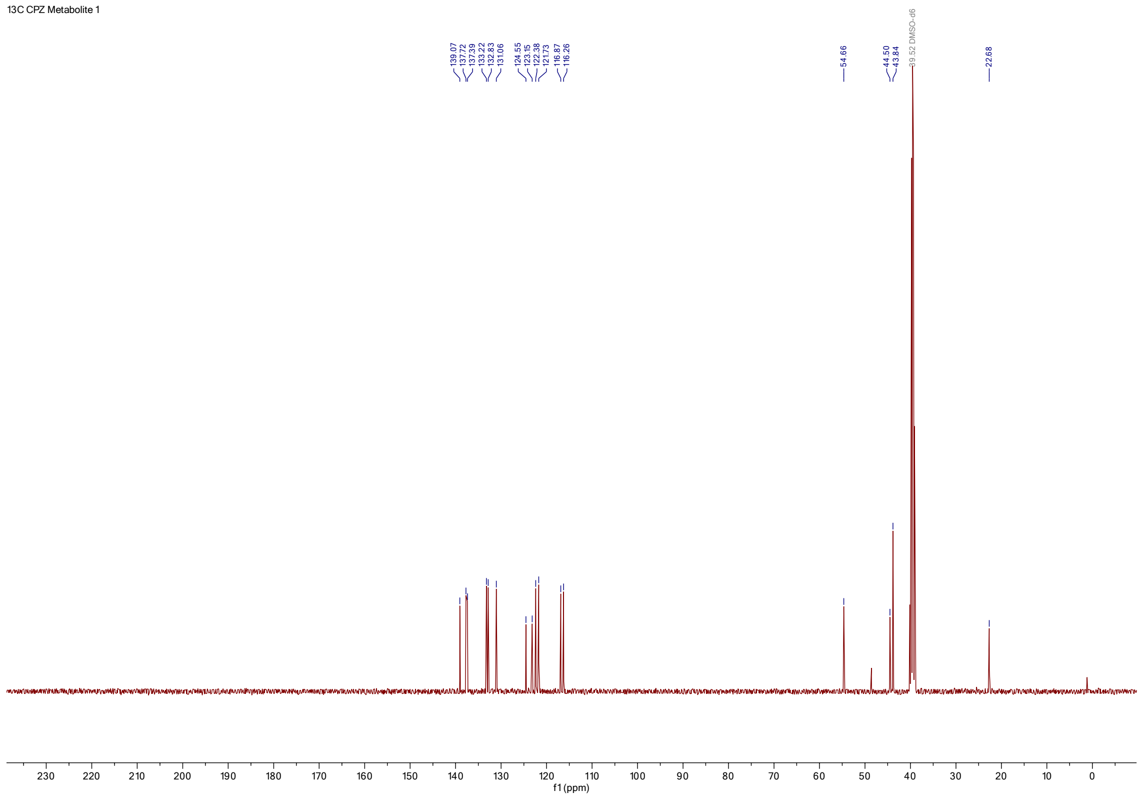

Figure S16. LRMS data for **CPZ-sulfoxide**

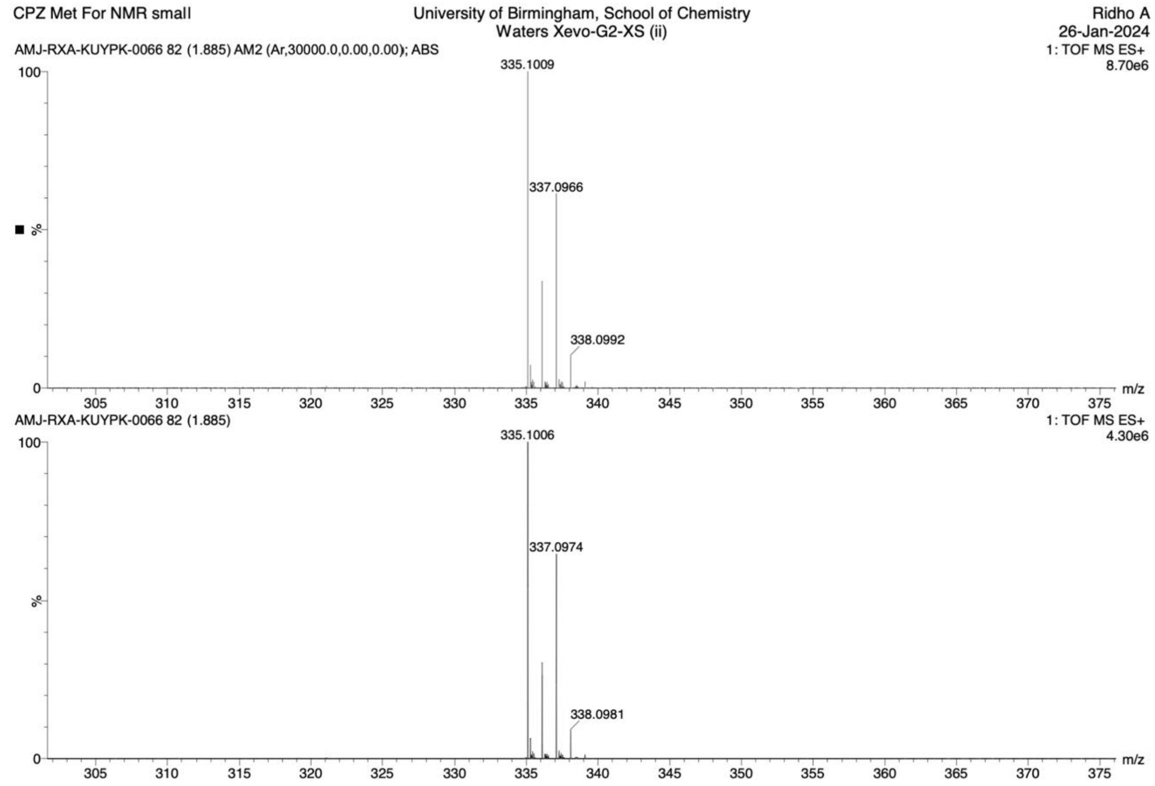

Figure S17. HRMS data for **CPZ-sulfoxide**

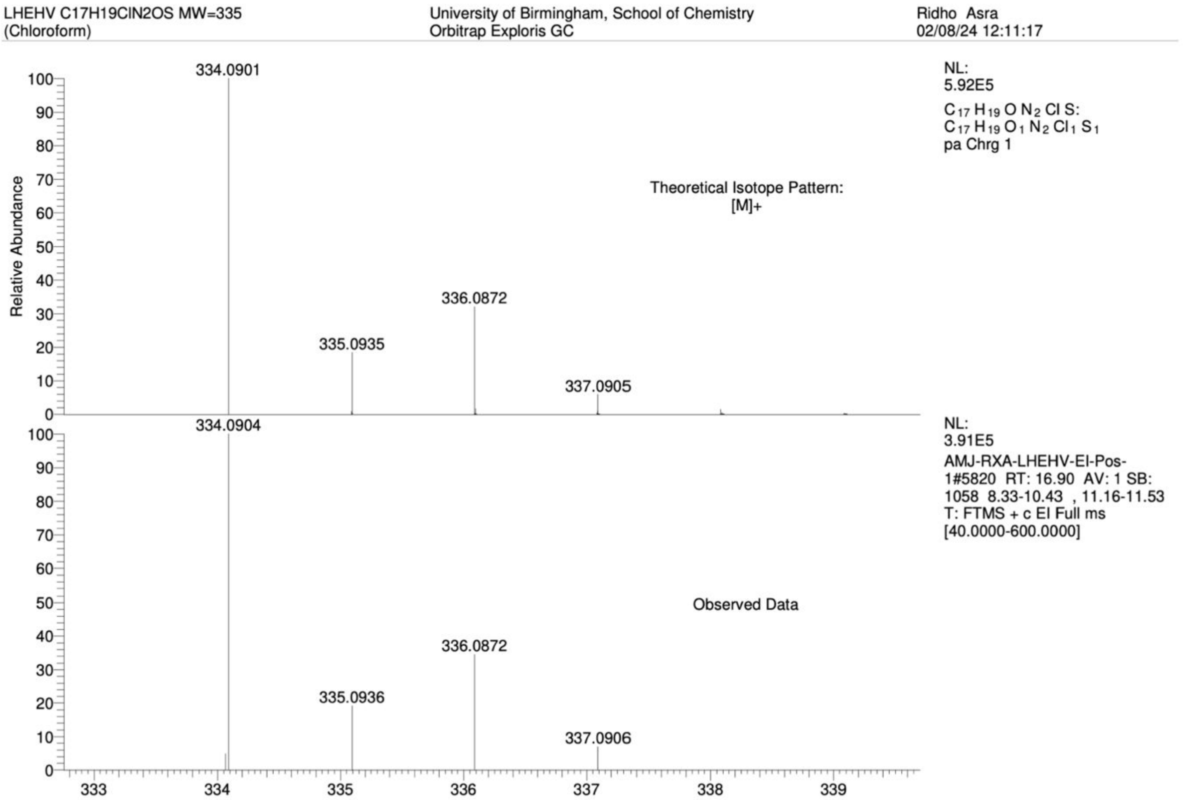

Figure S18. CPZ-sulfone (not isolated, detected by m/z)

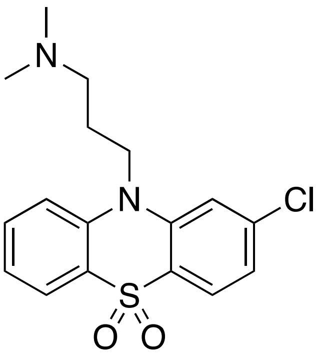

Figure S19. LRMS data for **CPZ-sulfone**

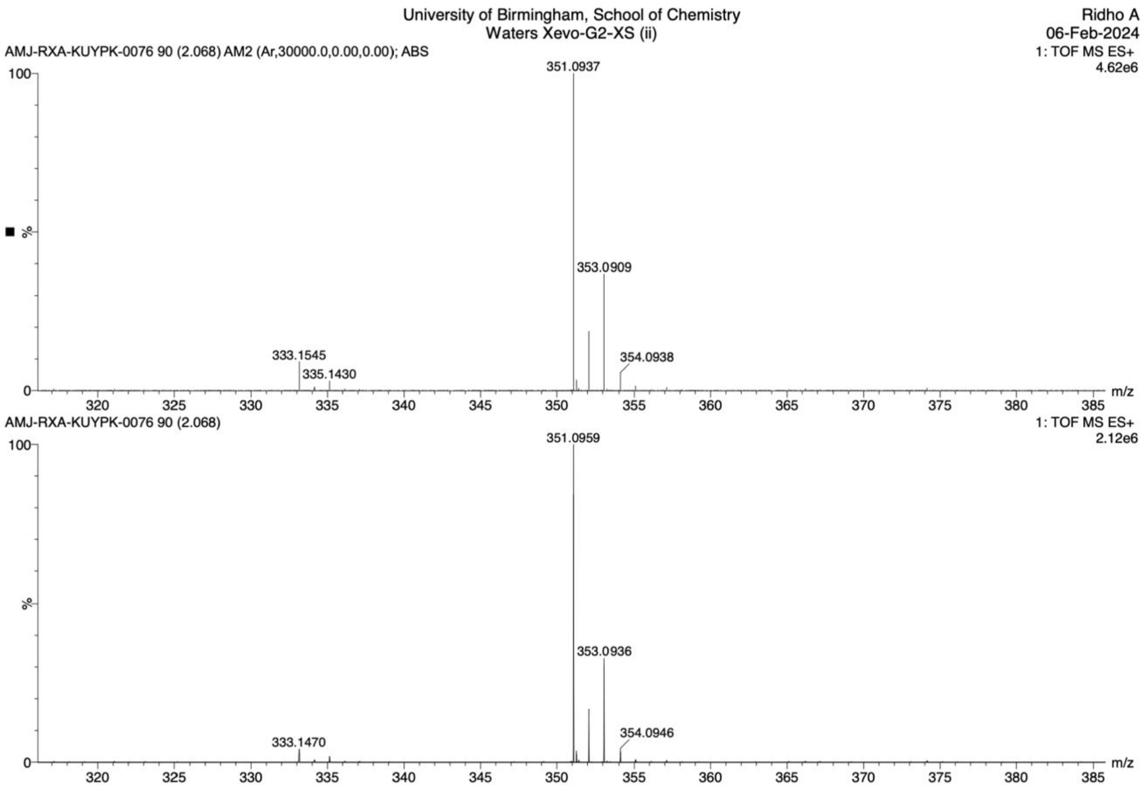

References

1. Bodoki, E.; Chira, R.; Zaharia, V.; Săndulescu, R., Mechanistic study of colchicine's electrochemical oxidation. *Electrochimica Acta* **2015,** *178*, 624-630.
2. Liu, K.; Meng, J.; Jiang, X., Gram-Scale Synthesis of Sulfoxides via Oxygen Enabled by Fe(NO3)3·9H2O. *Organic Process Research & Development* **2023,** *27* (7), 1198-1202.
